# Supplementary material for: Vibrational circular dichroism enhancement in conformationally flexible transition metal complexes
Source: Phys Chem Chem Phys. 2026 Sep 16;28(38):23655–63. doi: 10.1039/d6cp02625g (PMC13580204; doi:10.1039/d6cp02625g)
Supplement: Supplementary file 1 [file CP-028-D6CP02625G-s002.pdf]

Supplementary Information (SI) for Physical Chemistry Chemical Physics.
This journal is © the Owner Societies 2026

PCCP

ARTICLE TYPE

Cite this: DOI: 00.0000/xxxxxxxxxx

Supporting Information: Vibrational Circular Dichroism enhancement in conformationally flexible transition metal complexes

Mariia Sapova,[a] Menno de Boer,[a,b] Wybren Jan Buma,[c,d,*] and Lucas Visscher[a,*]

Table S1 Relative electronic and Gibbs free energies of the Λ-(R) and Δ-(R) conformers within a 6 kcal/mol energy window. Gibbs weights w_G were calculated at 298.15 K over the full set shown. The numbering of conformers indicates the energetic order predicted by the GOAT[1,2] conformer generation algorithm.

Conformer	E Eh	ΔE kcal mol^{-1}	G Eh	ΔG kcal mol^{-1}	w_G
Λ-(R)-conf 0	-3721.808690	0.639	-3721.381688	0.106	0.299
Λ-(R)-conf 1	-3721.809702	0.004	-3721.381219	0.400	0.182
Λ-(R)-conf 7	-3721.809090	0.388	-3721.380667	0.747	0.102
Λ-(R)-conf 11	-3721.809709	0.000	-3721.381857	0.000	0.358
Λ-(R)-conf 13	-3721.808228	0.929	-3721.380029	1.147	0.052
Λ-(R)-conf 14	-3721.803403	3.957	-3721.375731	3.844	0.001
Λ-(R)-conf 15	-3721.800655	5.681	-3721.373415	5.298	0.000
Λ-(R)-conf 16	-3721.800458	5.805	-3721.373198	5.433	0.000
Δ-(R)-conf 0	-3721.805905	2.387	-3721.377837	2.522	0.005
Δ-(R)-conf 6	-3721.802550	4.492	-3721.375018	4.292	0.000
Δ-(R)-conf 8	-3721.803521	3.883	-3721.375195	4.180	0.000
Δ-(R)-conf 19	-3721.801657	5.053	-3721.374848	4.398	0.000
Δ-(R)-conf 29	-3721.801857	4.927	-3721.373731	5.099	0.000
Δ-(R)-conf 30	-3721.802388	4.593	-3721.374830	4.410	0.000
Δ-(R)-conf 31	-3721.800278	5.918	-3721.372963	5.581	0.000
Δ-(R)-conf 36	-3721.802609	4.455	-3721.373863	5.016	0.000
Δ-(R)-conf 38	-3721.803113	4.139	-3721.374281	4.754	0.000
Δ-(R)-conf 44	-3721.801670	5.044	-3721.373280	5.382	0.000

[a] *Department of Chemistry and Pharmaceutical Sciences, Faculty of Sciences, Vrije Universiteit Amsterdam, De Boelelaan 1108, 1081 HZ Amsterdam, The Netherlands. E-mail: L.Visscher@vu.nl*
[b] *Theoretische Organische Chemie, Organisch-Chemisches Institut, Center for Multiscale Theory and Computation (CMTC), University of Münster, 48149 Münster, Germany.*
[c] *Van't Hoff Institute for Molecular Sciences, University of Amsterdam, Science Park 904, 1098 XH Amsterdam, The Netherlands. E-mail: W.J.Buma@uva.nl*
[d] *Radboud University, Institute for Molecules and Materials, FELIX Laboratory, Toernooiveld 7c, 6525 ED Nijmegen, The Netherlands*

Table S2 Vertical excitation energies (cm^{-1}) from TD-B3LYP[3] and CASSCF(7,5)/NEVPT2 calculations.

TD-B3LYP, Λ conformers								
#	Λ-conf 0	Λ-conf 1	Λ-conf 7	Λ-conf 11	Λ-conf 13	Λ-conf 14	Λ-conf 15	Λ-conf 16
1	6340	6364	6622	6202	6509	6509	6412	6452
2	9913	9784	9590	9703	9961	9719	9477	9429
3	10308	10251	10816	10171	11090	10396	10493	10679
4	11977	12098	11937	12227	11743	12518	12582	12179
5	16131	15913	17389	15712	17623	15841	16228	16567
6	18873	19027	18123	19059	17833	19422	19511	19075
TD-B3LYP, Δ conformers								
#	Δ-conf 0	Δ-conf 6	Δ-conf 8	Δ-conf 19	Δ-conf 29	Δ-conf 30	Δ-conf 31	Δ-conf 36
1	6646	6743	6348	6493	6356	6331	6638	6235
2	10477	9945	9767	10380	9429	9775	10138	8824
3	11155	11155	10251	10711	10655	10606	11268	10179
4	11477	11493	12671	11872	11832	12171	11469	12687
5	17293	17438	15913	16865	16058	16252	17696	14849
6	17728	17881	19252	18349	18970	19115	17825	19994
#	Δ-conf 38	Δ-conf 44						
1	6275	6017						
2	9082	8727						
3	10235	10001						
4	12873	13413						
5	15187	14857						
6	19986	20712						
CASSCF/NEVPT2								
#	Λ-conf 11	Δ-conf 0						
1	2354	2602						
2	6068	7397						
3	8303	7742						
4	8784	9440						
5	9519	9670						
6	10053	10098						

Λ-(R)-conf 0

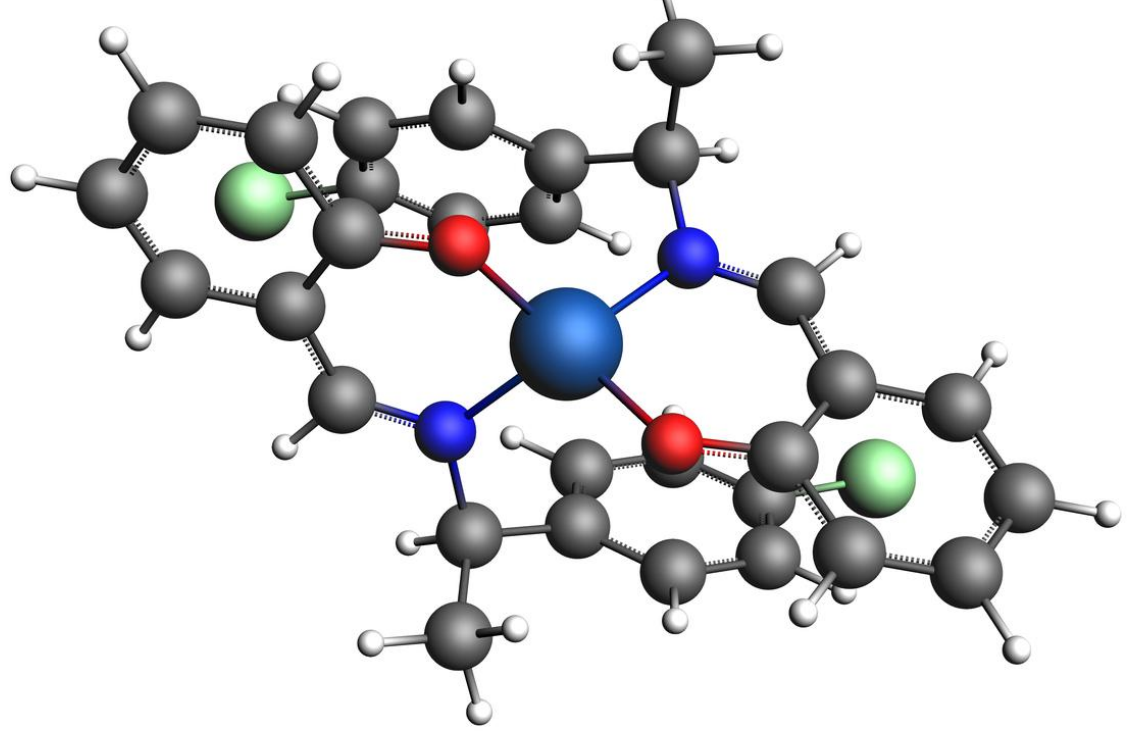

Λ-(R)-conf 1

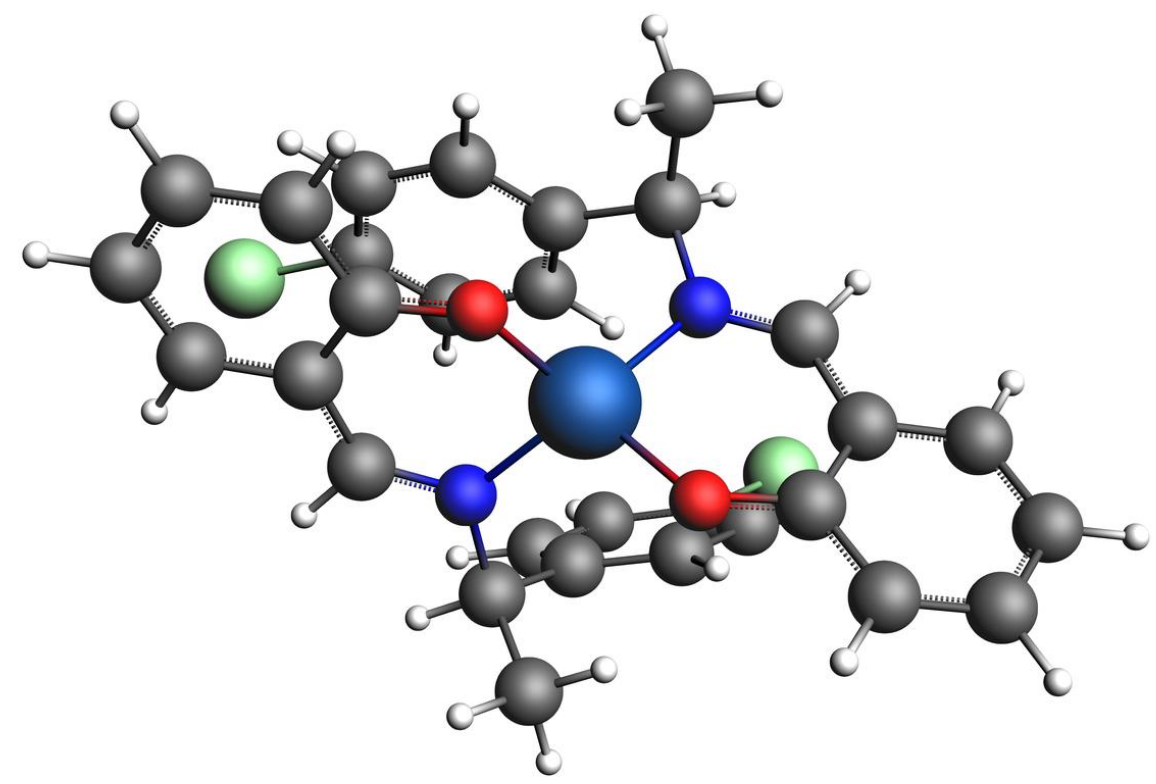

Λ-(R)-conf 7

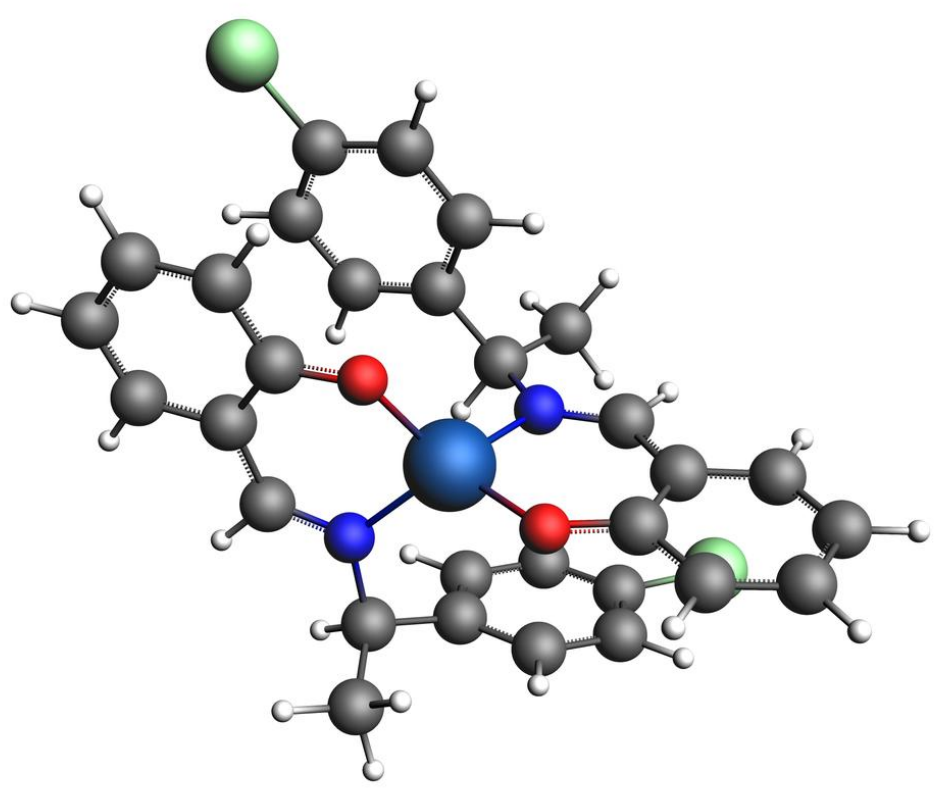

Λ-(R)-conf 11

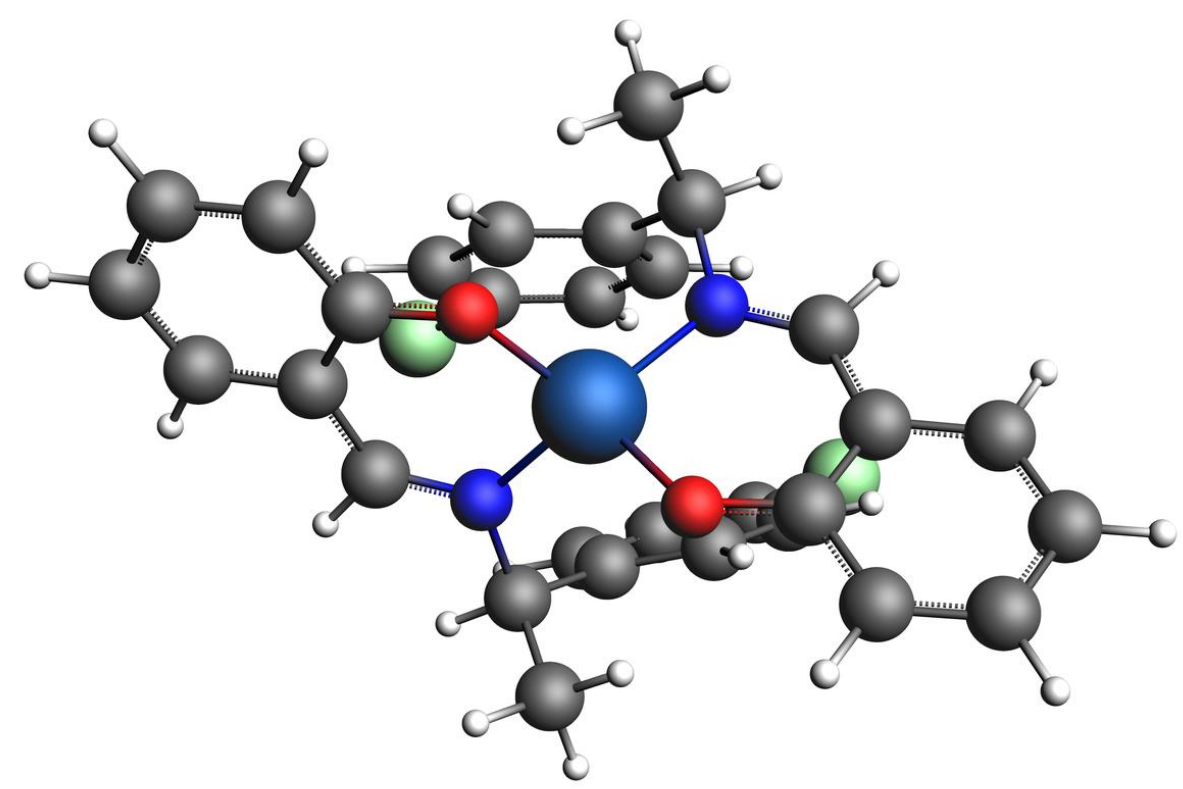

Λ-(R)-conf 13

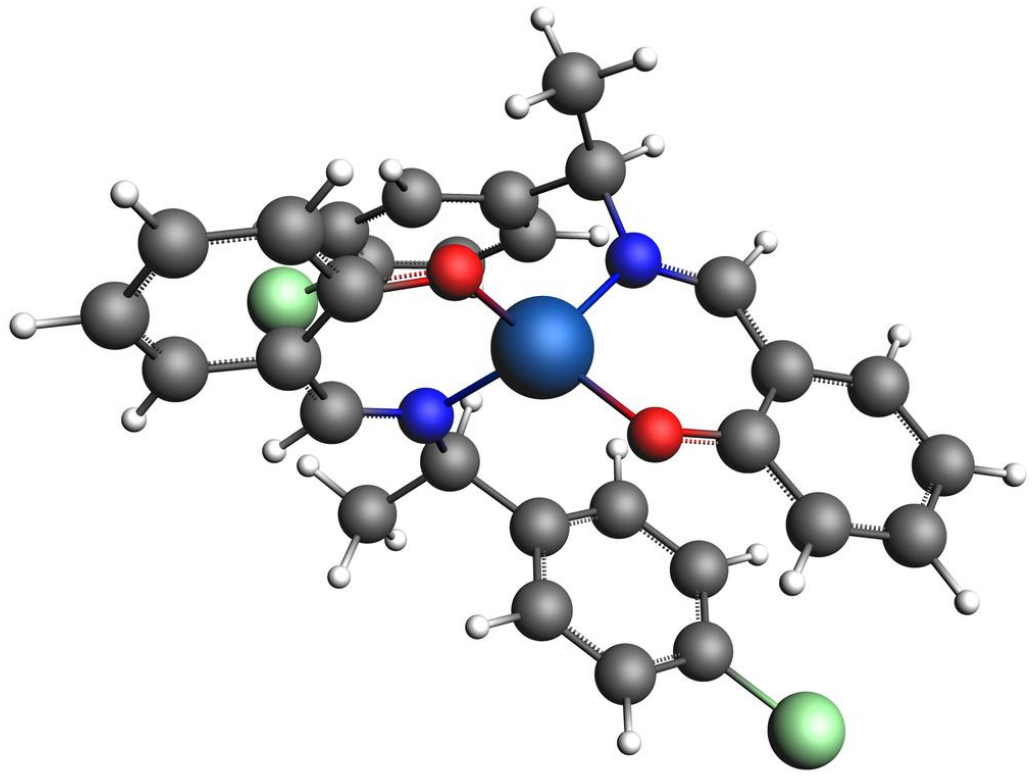

Λ-(R)-conf 14

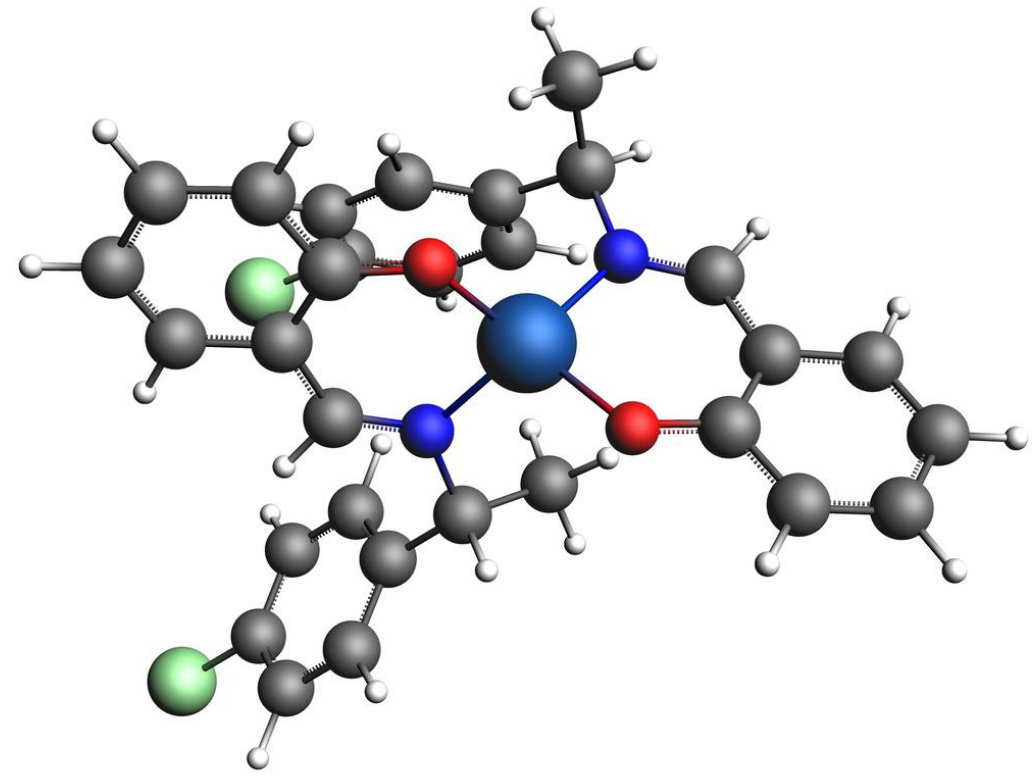

Λ-(R)-conf 15

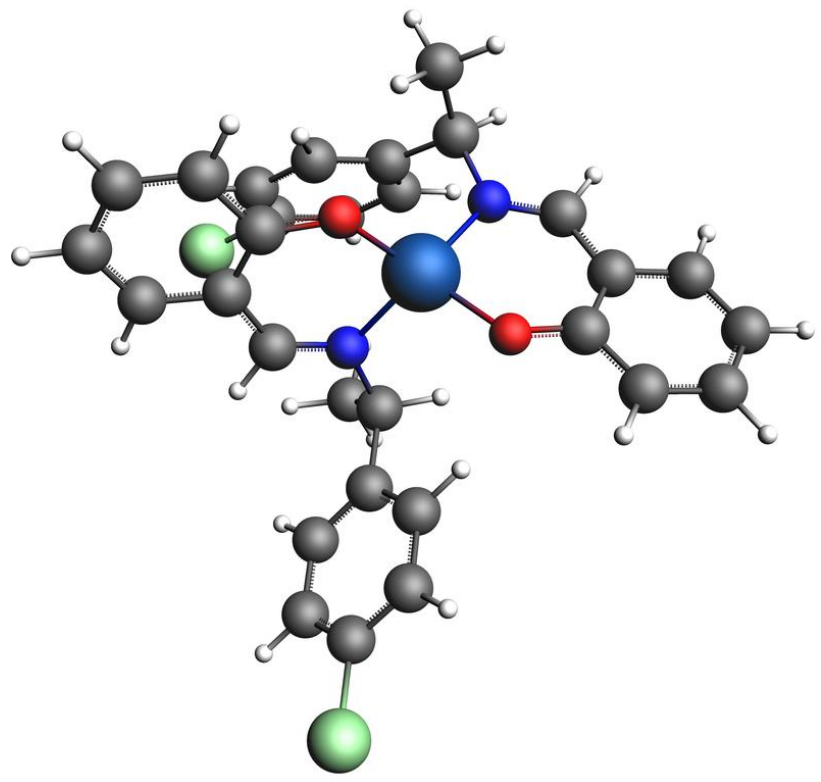

Λ-(R)-conf 16

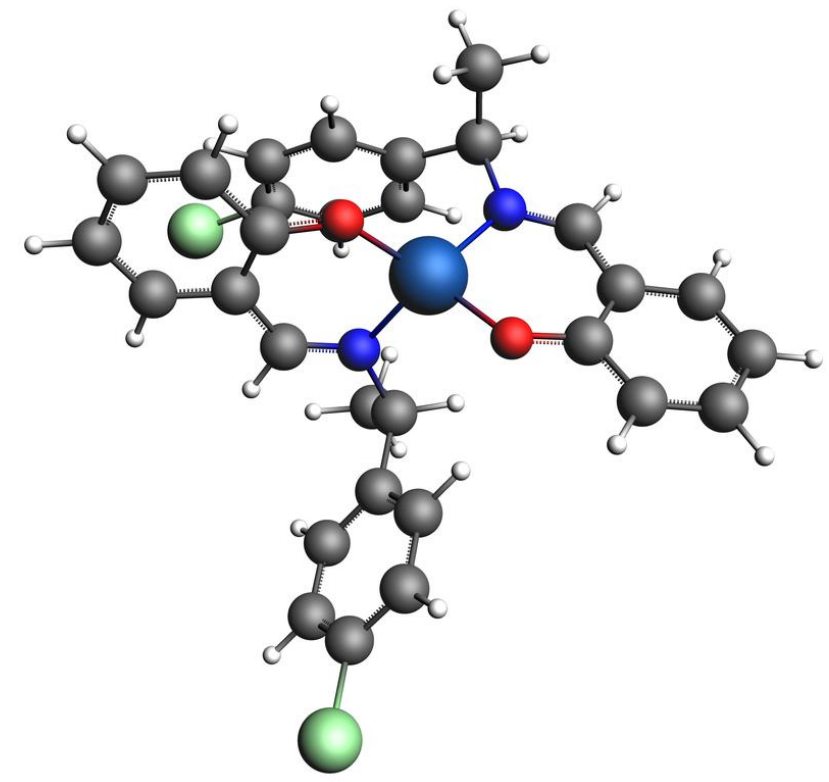

Fig. S1 Λ conformer structures within 6 kcal/mol energy window. Λ-(R)-conf 11 is the lowest lying conformer.

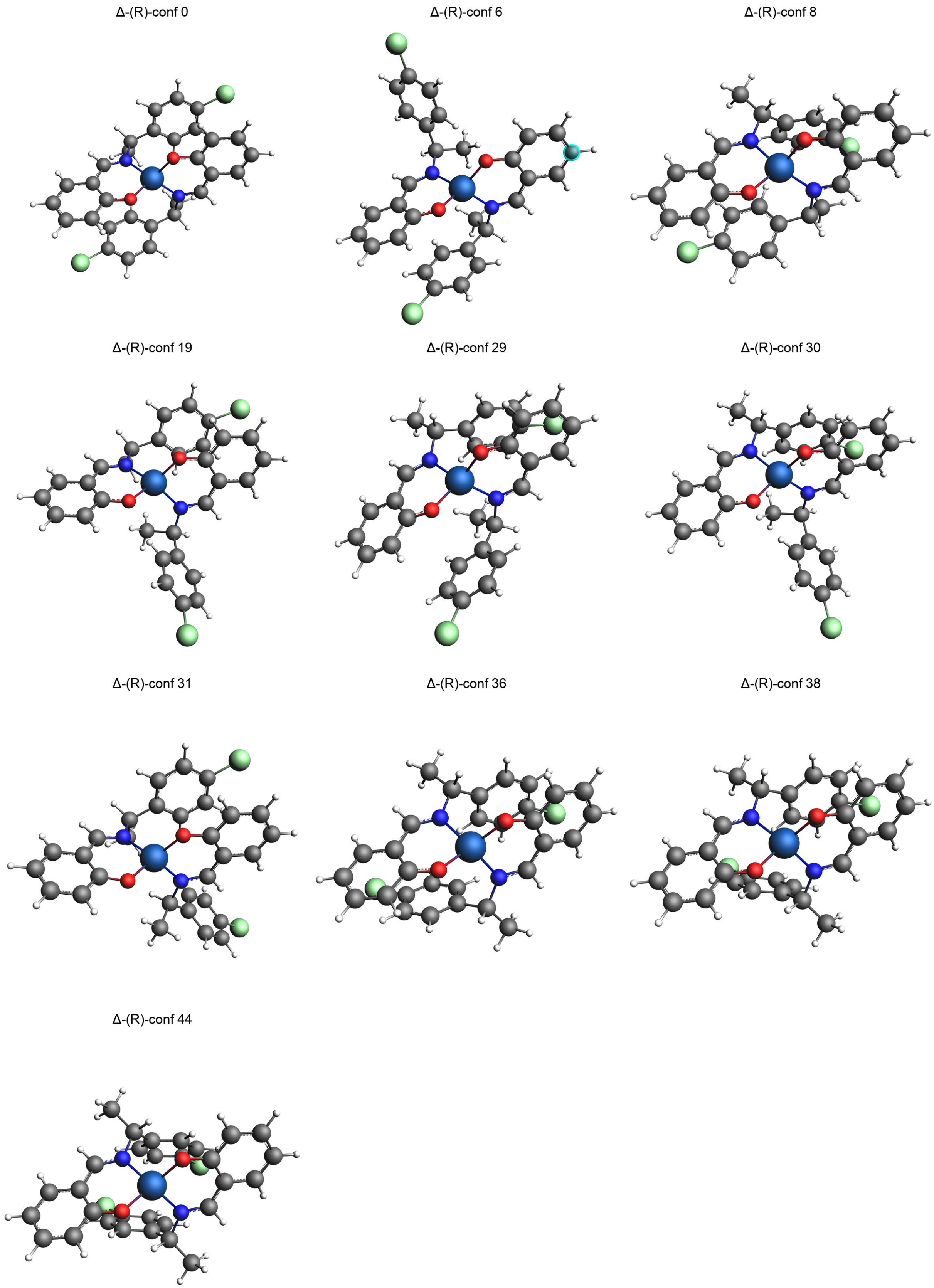

Fig. S2 Δ conformer structures within 6 kcal/mol energy window.

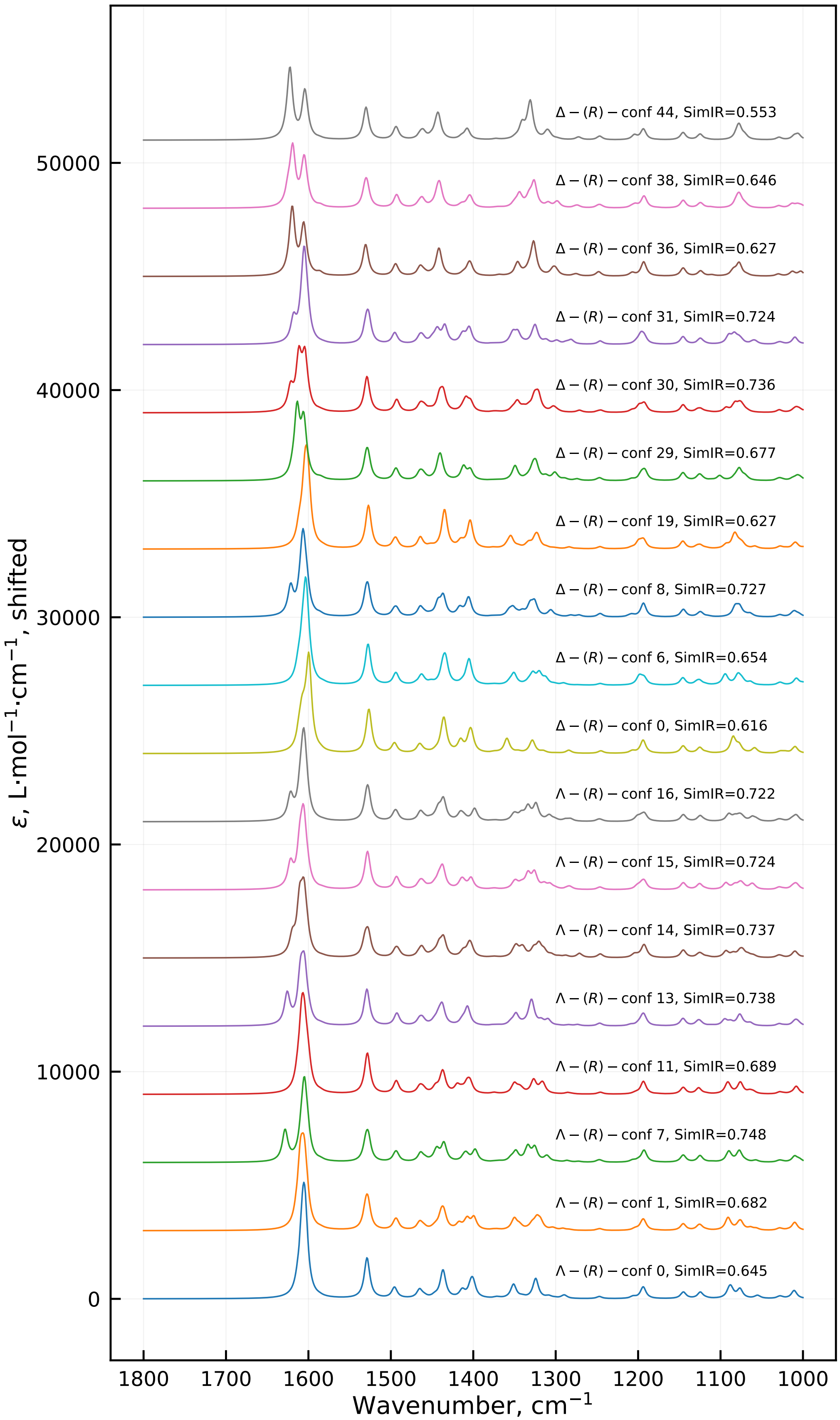

Fig. S3 IR spectra of unique conformers calculated with B3LYP-D3BJ/def2-TZVP/CPCM.

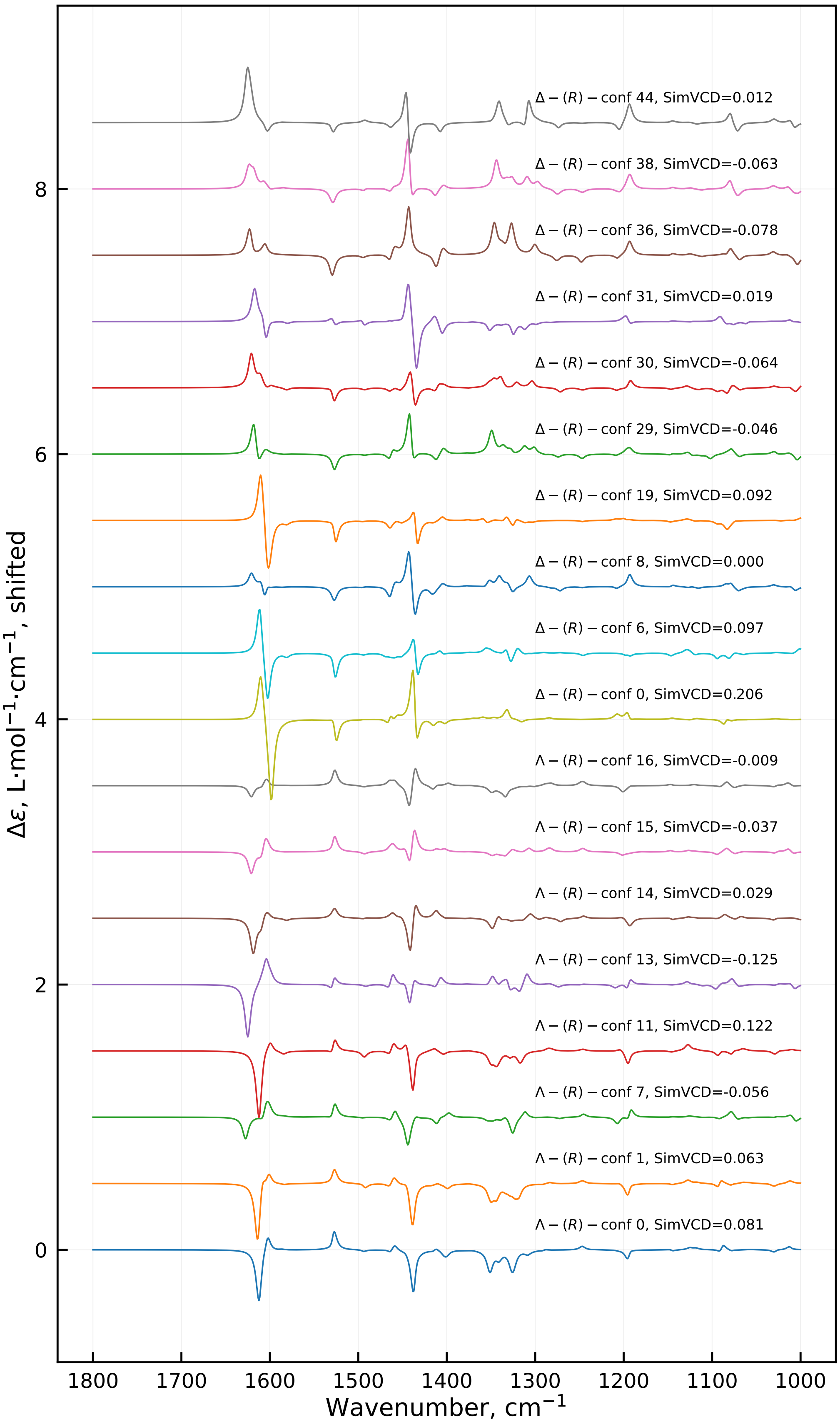

Fig. S4 MFP-VCD spectra of unique conformers calculated with B3LYP-D3BJ/def2-TZVP/CPCM.

1 Enhanced VCD calculation

In the enhanced VCD calculations we include coupling only to the first electronically excited state. This choice is supported by a significant gap between the first and higher electronically excited states obtained both at the TDDFT and CASSCF/NEVPT2 level (Table S2). The second and third excited states, in contrast, are close in energy and change order for some conformers. Because our model assigns the same excitation energies to all conformers, the same electronic state must be followed consistently across the conformer ensemble. In the present system, these higher states make only a minor contribution to the enhancement, so the first excited state provides a compact and quantitative description of the effect.

We then compared enhanced VCD fits obtained with the full conformer set and with a reduced set in which spectrally redundant conformers were removed. In both cases, the fit parameters were the conformer energy shifts within a chosen uncertainty range and a single shared excitation energy. We tested conformer energy uncertainty ranges of ±1, ±2, and ±3 kcal mol^{-1} (Figure S5). Increasing the uncertainty from ±2 to ±3 kcal mol^{-1} was found to only have a minor effect on the optimized spectra and the Λ:Δ ratio (see simVCD and Λ:Δ in Figure S5). A potential concern is that the intense C=N stretching region near 1600 cm^{-1} dominates the SimVCD metric. This band is also overestimated in the calculated IR spectrum, suggesting that the corresponding VCD intensity may be overestimated as well. Therefore, fitting the full spectral range can improve the apparent similarity by reproducing this dominant feature while giving less reliable agreement in the lower-frequency region. To test this effect, we evaluated the spectral similarity both over the full range and with the dominant C=N feature excluded. For the reduced model, the full-range SimVCD increases, but the similarity outside the C=N region does not increase correspondingly. This indicates that this improvement is driven mainly by the intense C=N band rather than by a uniformly better description of the spectrum. Therefore, we excluded the C=N-dominated region from the fit while still reporting the final similarity over the full spectral range (Figure S6). With this more balanced fitting procedure, the full and reduced conformer sets give very similar results. The difference in SimVCD is only about 0.03, and visual inspection concludes that removing 10 spectrally redundant conformers does not qualitatively change the spectrum. The fitted Λ:Δ ratio also becomes close to 70:30, in better agreement with the experimental ratio of approximately 80:20.

To investigate whether an even smaller conformer set is sufficient to describe the enhanced VCD spectrum, we fitted the excitation energy for a single Λ-conf 11 excluding C=N dominated region (Figure S7). This single-conformer model reproduces the main negative features of the experimental spectrum already reasonably well and leads to a fitted excitation energy of 2499 cm^{-1}. Including a lowest-energy Δ conformer (Δ-conf 0) markedly improves the spectra similarity from 0.565 for the single-conformer model to 0.740 for two-conformer model and an excitation energy of 2391.8 cm^{-1}. This result remains consistent with what is experimentally observed in terms of transitions to low-lying electronically excited states. Finally, we compared the Λ:Δ ratio for three considered conformer sets: (i) 18 conformers within a 6 kcal/mol energy window, (ii) 8 spectrally independent conformers and (iii) the minimal two-conformer set. The resulting ratios are only slightly different: 0.69:0.31, 0.73:0.27 and 0.68:0.32. Thus, the enhanced VCD spectrum is primarily sensitive to the overall Λ/Δ balance rather than to the detailed distribution among individual conformers.

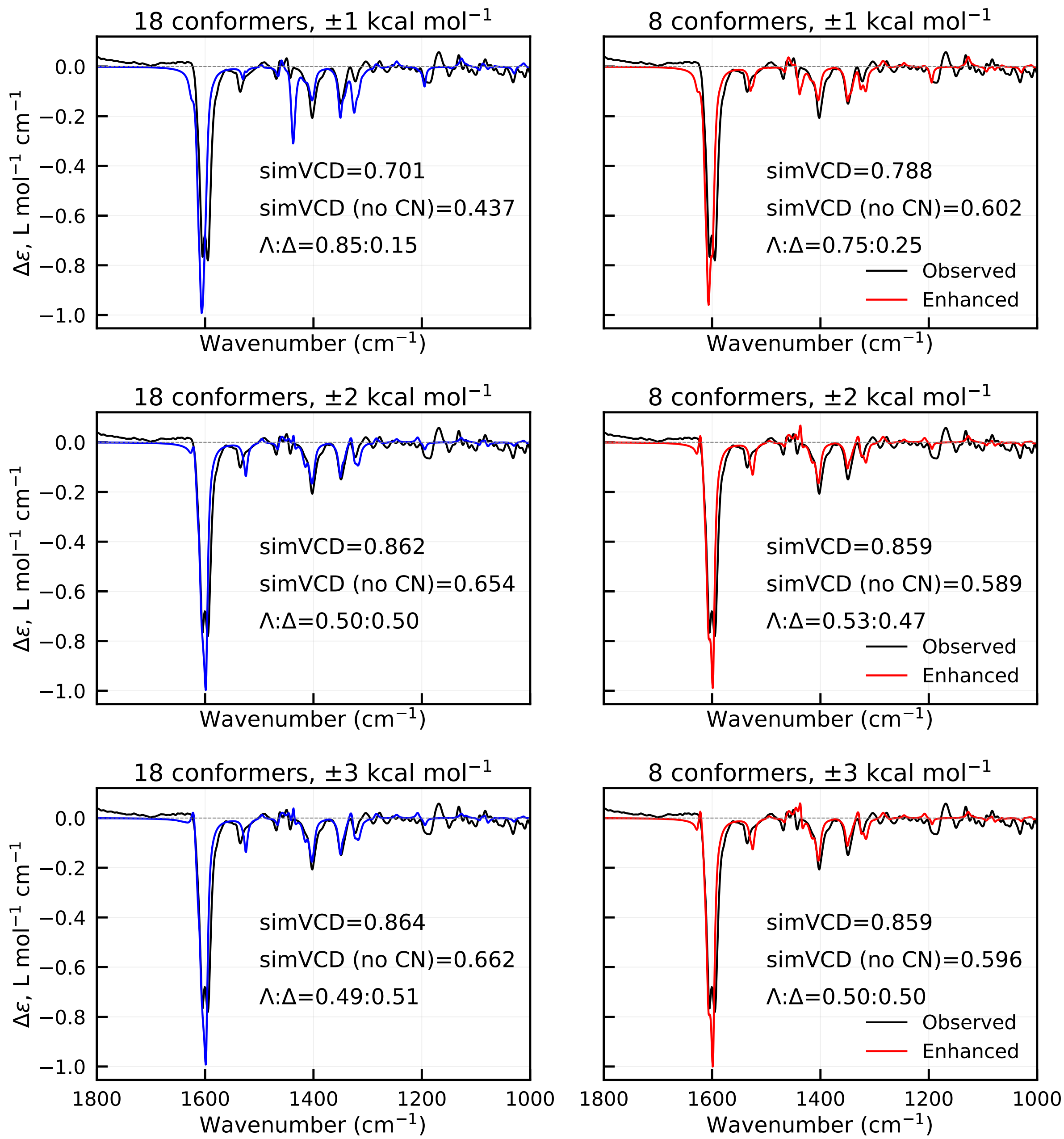

Fig. S5 Enhanced VCD spectra calculated with all unique conformers within a 6 kcal/mol energy window (left) and with spectrally unique conformers (right) fitted the full 1000-1800 cm^{-1} region. SimVCD values are calculated on the full frequency range, simVCD (no CN) excludes dominating C=N peaks.

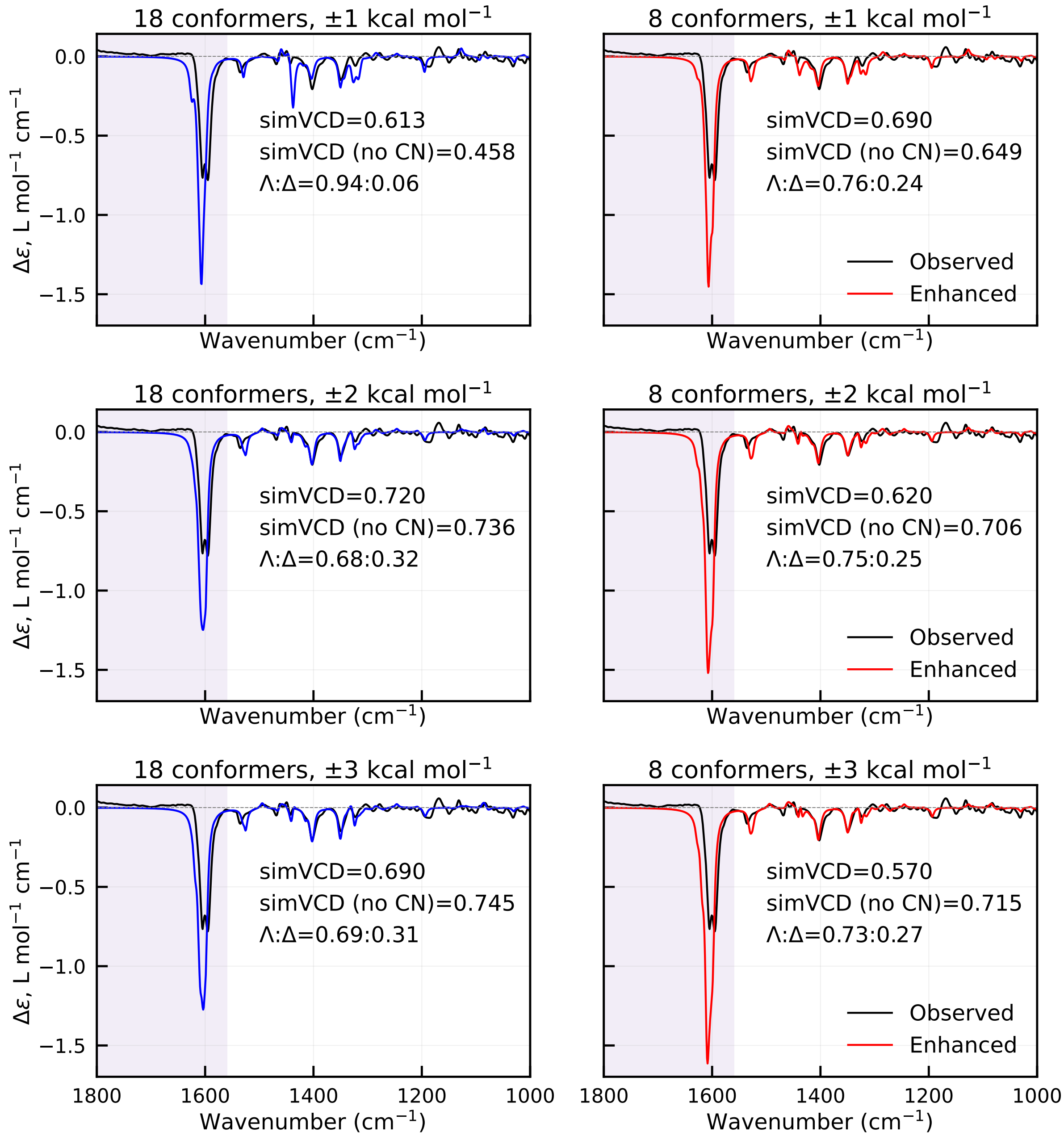

Fig. S6 Enhanced VCD spectra calculated with all unique conformers within a 6 kcal/mol energy window (left) and with spectrally unique conformers (right) resulting from fits in which the purple range has been excluded.

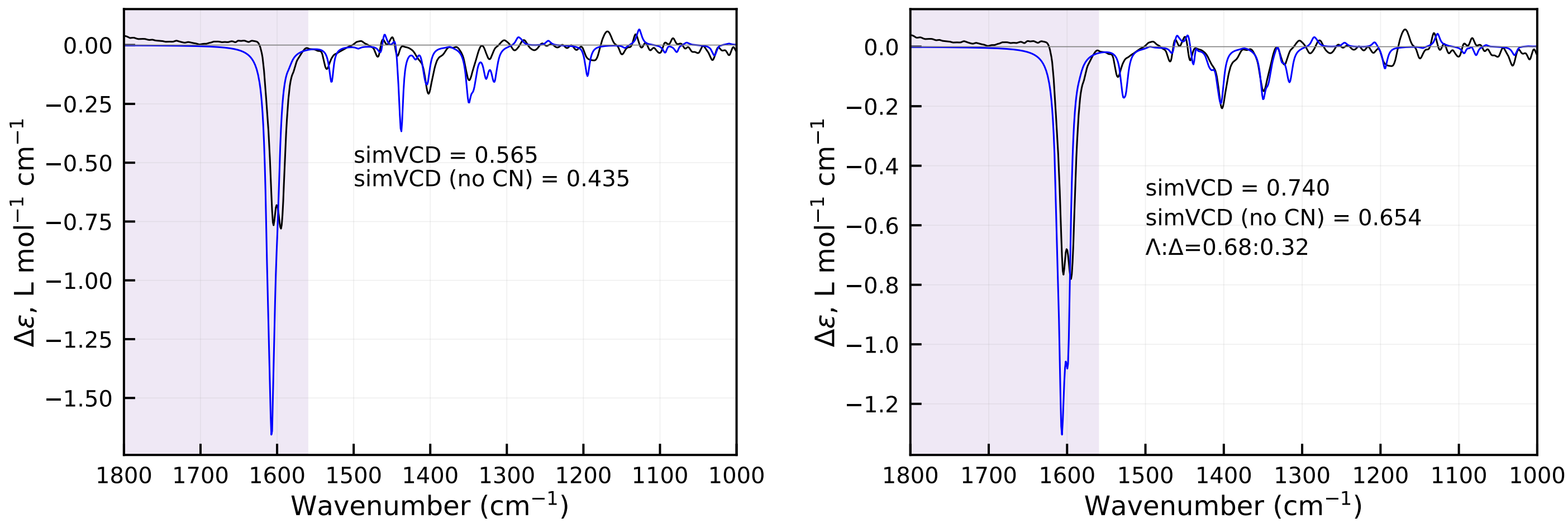

Fig. S7 Enhanced VCD spectra calculated with single conformer Λ-conf 11 (left), and with two conformers (Λ-conf 11 and Δ-conf 0) (right).

1.1 Calculations without dispersion correction and solvent model

We performed additional B3LYP[3]/def2-TZVP[4] calculations to reproduce the level of theory used in[5]. At this level we reoptimized 26 Λ and 51 Δ structures generated by GOAT[1,2]. Consistent with previous observations[6], the lowest-energy structure has Δ metal center chirality, contrary to the experimental preference for the Λ form. However, our DFT reoptimization yielded a larger set of unique structures: 9 Λ and 11 Δ conformers within 5 kcal mol^{-1}. All of these structures were included in the enhanced VCD calculation. The agreement with experiment is poorer than that for the conformer set optimized with dispersion correction and the solvent model. Nevertheless, allowing uncertainty in the Gibbs free energies ($\pm$3 kcal mol^{-1}) increases the Λ population, opposite to the raw DFT Boltzmann weights and leads to qualitative agreement with the experimental data. The optimized model gives a Λ:Δ ratio of 63:37 and an excitation energy of 2203 cm^{-1}, with SimVCD = 0.595 for the full range and SimVCD = 0.566 when the C=N region is excluded (see Figure S8).

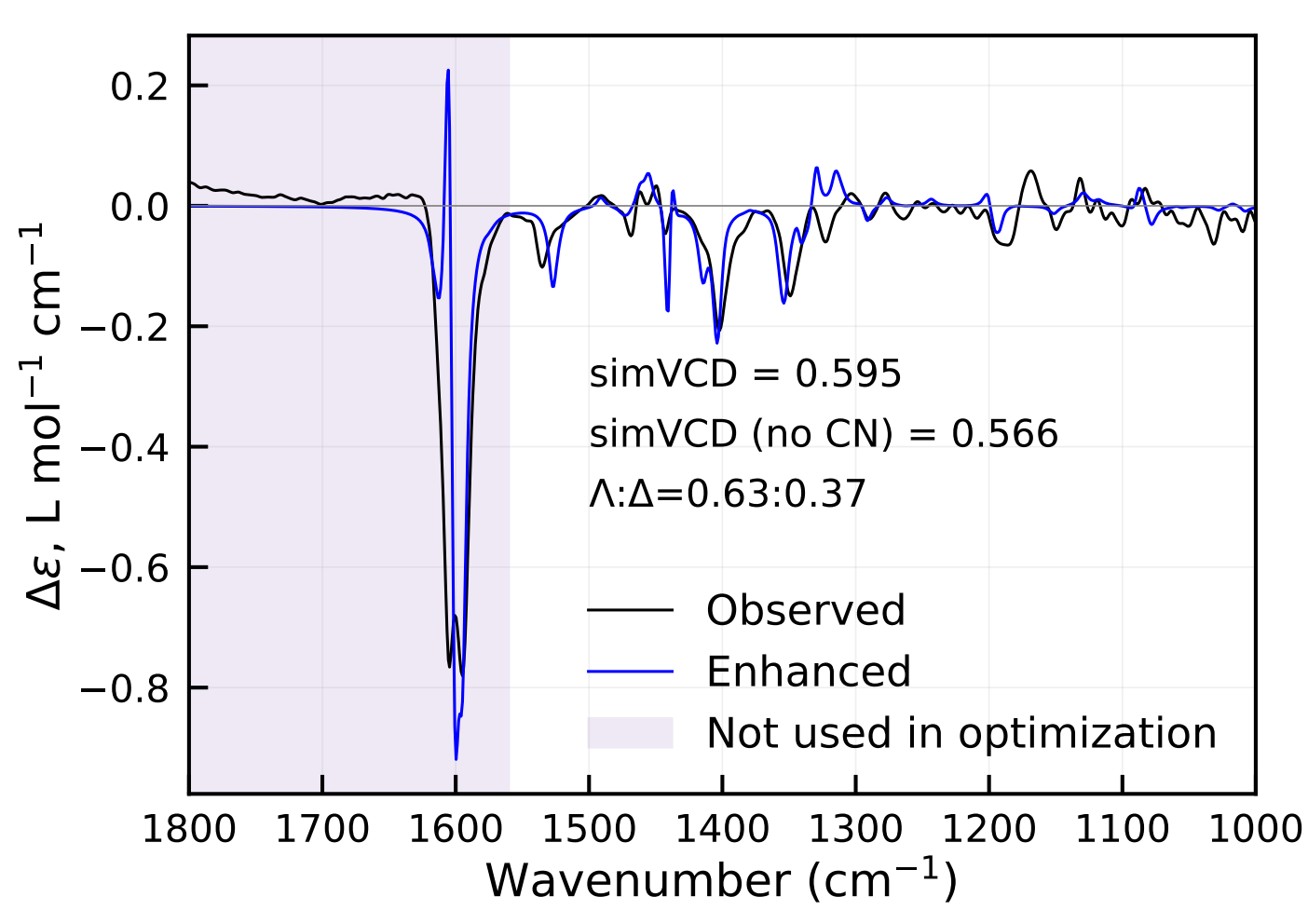

Fig. S8 Enhanced VCD spectra calculated with all unique conformers optimized on B3LYP/def2-TZVP level.

1.2 Other aryl groups

For other substituents (X = H, OCH_3,Br) we used the two-conformer model and the B3LYP-D3BJ/def2-TZVP/CPCM level of theory. We reoptimized conformers Λ-conf 11 and Δ-conf 0 conformers. Figure S9 to S11 show that the two considered conformers describe the enhanced VCD spectra well, although separately optimized conformer sets are probably needed for more reliable estimates of the excitation energy and Λ population distribution. Similarity values over the full ranges are 0.674 for H, 0.817 for the OCH_3, and 0.754 for the Br substituent.

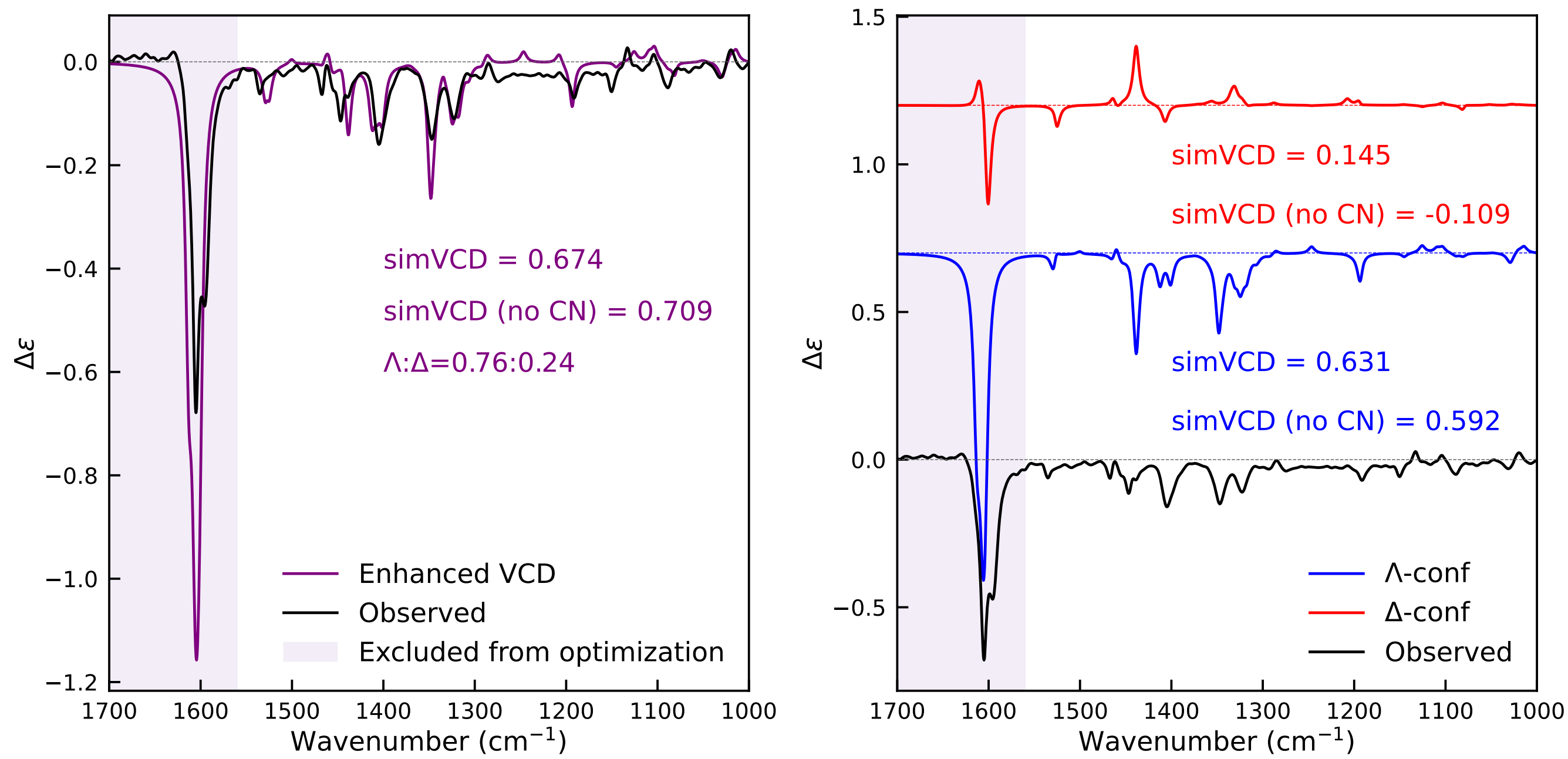

Fig. S9 Enhanced VCD spectra of averaged over two Λ and Δ conformers (left) for H as substituent. Individual weighted contributions of the two conformer with the SimVCD values (right). The fitted excitation energy is 2516 cm^{-1}.

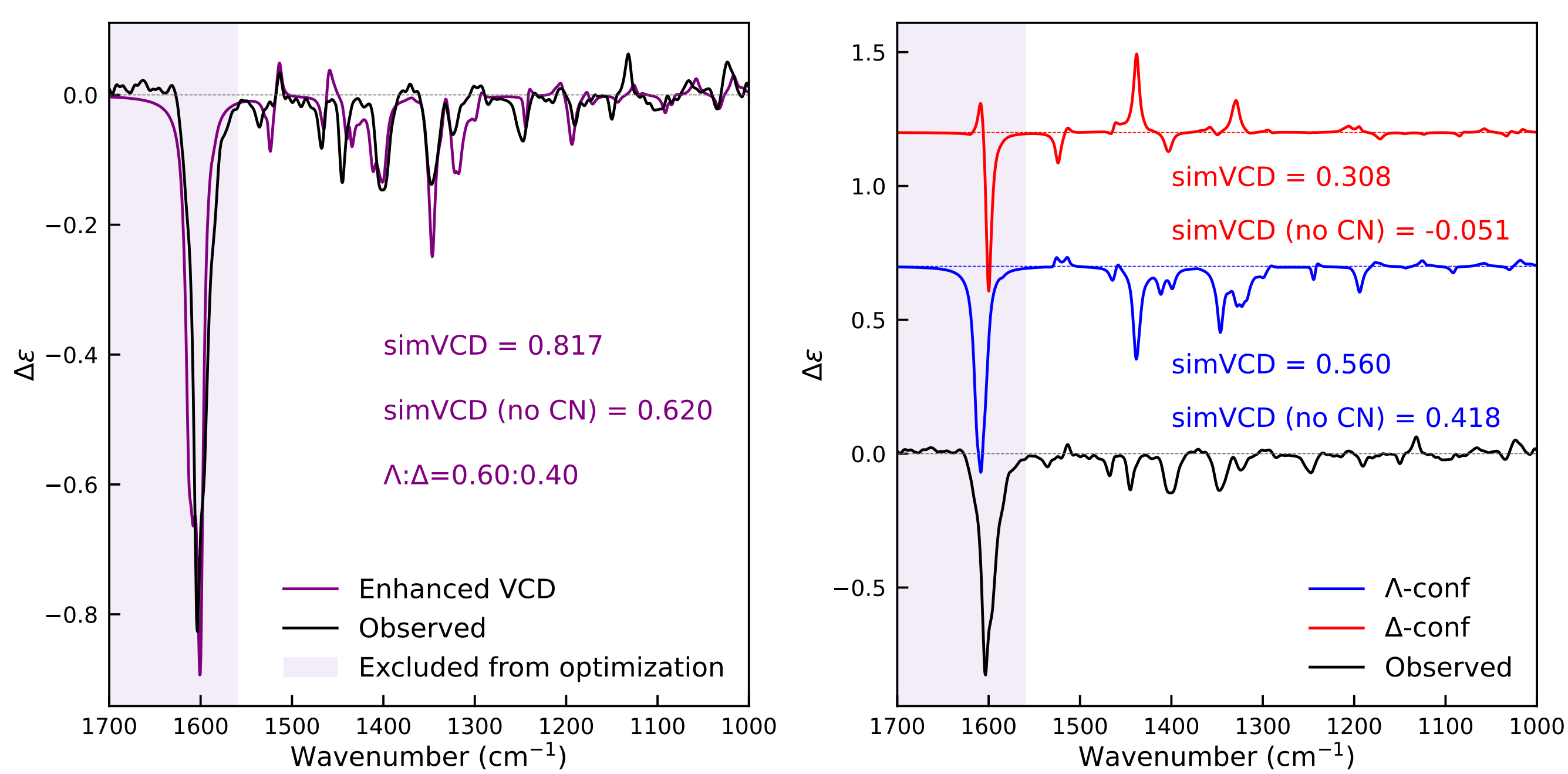

Fig. S10 Enhanced VCD spectra averaged over two Λ and Δ conformers (left) for OCH_3 as substituent. Individual weighted contributions of the two conformer with the SimVCD values (right). The fitted excitation energy is 2588 cm^{-1}.

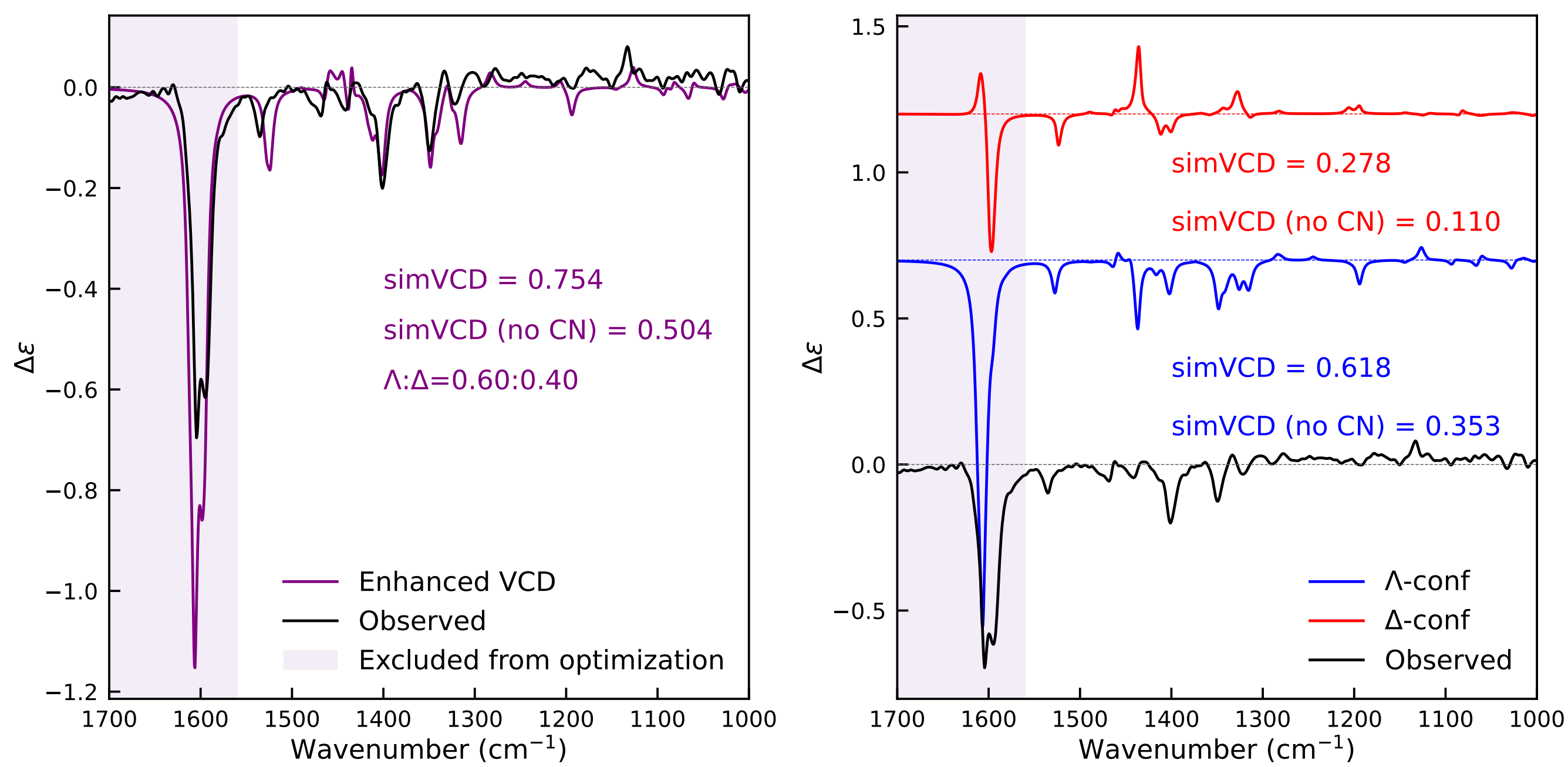

Fig. S11 Enhanced VCD spectra averaged over two Λ and Δ conformers (left) for Br as substituent. Individual weighted contributions of the two conformer with the SimVCD values (right). The fitted excitation energy is 2403 cm^{-1}.

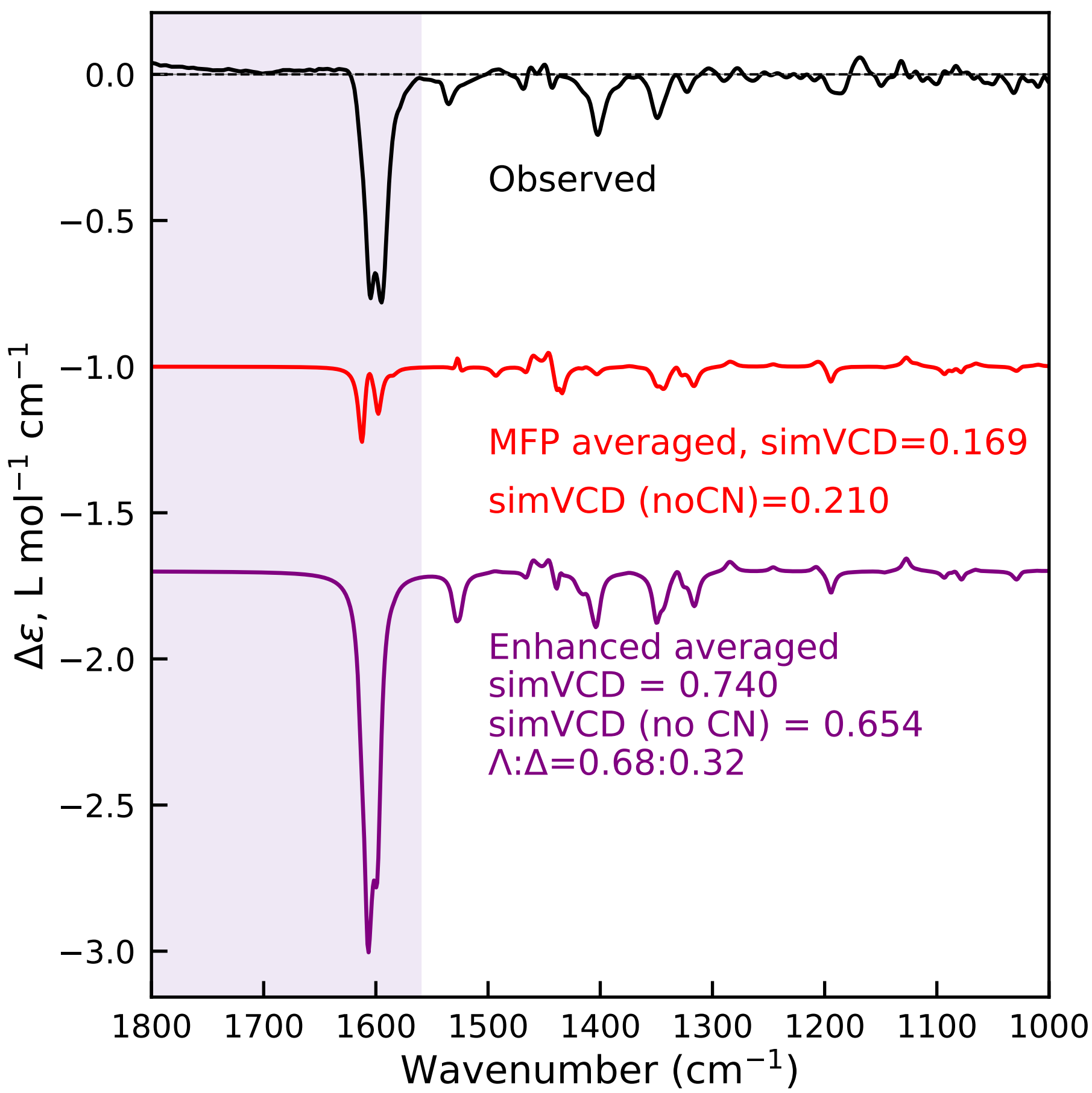

Fig. S12 Enhanced VCD spectra averaged over two Λ and Δ conformers for Cl substituent. The spectrum shows the difference between calculated MFP and enhanced VCD spectra. We use conformer populations from enhanced spectrum fitting for a direct comparison.

2 Symmetry analysis

Table S3 Symmetry-allowed MFP rotational-strength components and enhancement transitions for chiral point groups C_n, D_n, T and O (n = 1-6). It indicates which electronic transition can enhance the vibrational modes of particular symmetry and through which Cartesian component of the dipole. For point groups with only proper rotations electric and magnetic dipole moment components transform in the same manner.

Point group	VCD/IR Active Mode	Cartesian component	Enhancement transition
C_1	A	x,y,z	$A \to A$
C_2	A	z	$A \to A$, $B \to B$
	B	x,y	$A \to B$
C_3	A	z	$A \to A$, $E \to E$
	E	x,y	$A \to E$
C_4	A	z	$A \to A$, $B \to B$, $E \to E$
	E	x,y	$A \to E$, $B \to E$
C_5	A	z	$A \to A$, $E_1 \to E_1$,$E_2 \to E_2$
	E_1	x,y	$A \to E_1$, $E_1 \to E_2$, $E_2 \to E_2$
C_6	A	z	$A \to A$, $B \to B$, $E_1 \to E_1$, $E_2 \to E_2$
	E_1	x,y	$A \to E_1$, $B \to E_2$, $E_1 \to E_2$
D_2	B_1	z	$A \to B_1$, $B_2 \to B_3$
	B_2	y	$A \to B_2$, $B_1 \to B_3$
	B_3	x	$A \to B_3$, $B_1 \to B_2$
D_3	A_2	z	$A_1 \to A_2, E \to E$
	E	x,y	$A_1 \to E$, $A_2 \to E$, $E \to E$
D_4	A_2	z	$A_1 \to A_2, B_1 \to B_2, E \to E$
	E	x,y	$A_1, A_2, B_1, B_2 \to E$,
D_5	A_2	z	$A_1 \to A_2$, $E_1 \to E_1, E_2 \to E_2$
	E_1	x,y	$A_1, A_2, E_2 \to E_1$
D_6	A_2	z	$A_1 \to A_2$, $E_1 \to E_1$
	E_1	x,y	$A_2 \to E_1$
T	T	x,y,z	$A_1, E_1, E_2, T \to T$
O	T_1	x,y,z	$A_1, E, T_1, T_2 \to T_1$; $A_2, E, T_2 \to T_2$

Notes and references

1 F. Neese, *WIREs Computational Molecular Science*, 2025, **15**, e70019.

2 B. de Souza, *Angewandte Chemie International Edition*, 2025, **64**, e202500393.

3 A. D. Becke, *The Journal of chemical physics*, 1993, **98**, 5648–5652.

4 F. Weigend and R. Ahlrichs, *Physical Chemistry Chemical Physics*, 2005, **7**, 3297–3305.

5 G. Pescitelli, S. Lüdeke, M. Górecki and L. Di Bari, *The Journal of Physical Chemistry Letters*, 2019, **10**, 650–654.

6 G. Pescitelli, S. Lüdeke, A.-C. Chamayou, M. Marolt, V. Justus, M. Górecki, L. Arrico, L. Di Bari, M. A. Islam, I. Gruber, M. Enamullah and C. Janiak, *Inorganic Chemistry*, 2018, **57**, 13397–13408.
