# Supplementary material for: Vibrational circular dichroism enhancement in conformationally flexible transition metal complexes
Source: Phys Chem Chem Phys. 2026 Sep 16;28(38):23655–63. doi: 10.1039/d6cp02625g (PMC13580204; doi:10.1039/d6cp02625g)

Δ-(R)-conf 0

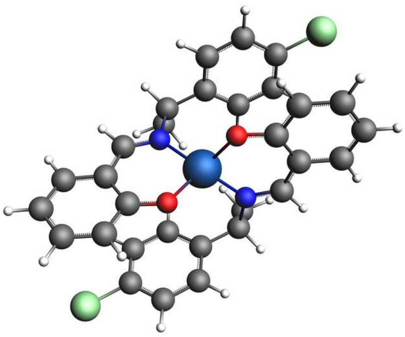

Δ-(R)-conf 6

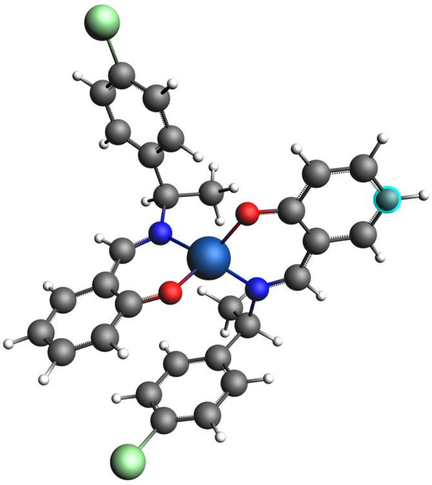

Δ-(R)-conf 8

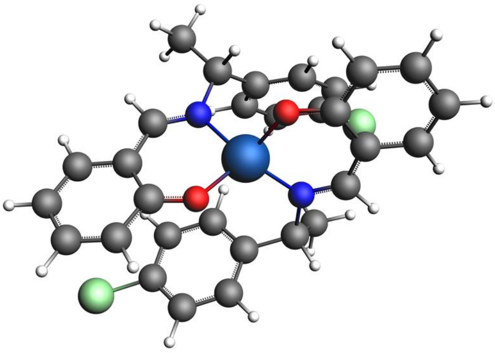

Δ-(R)-conf 19

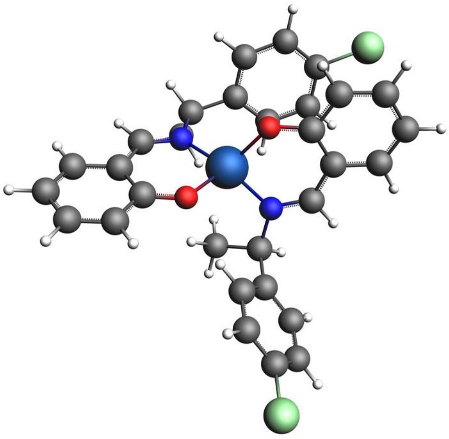

Δ-(R)-conf 29

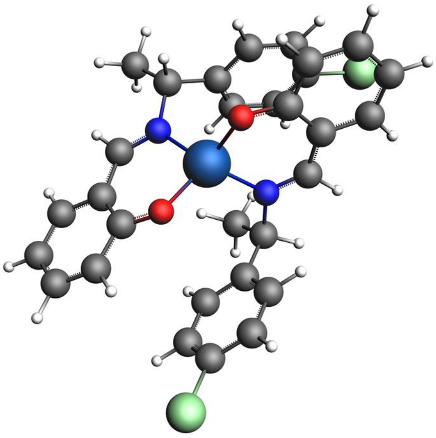

Δ-(R)-conf 30

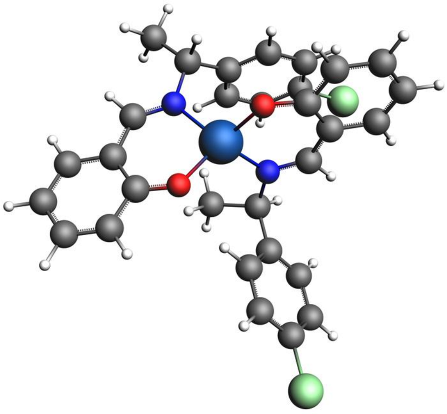

Δ-(R)-conf 31

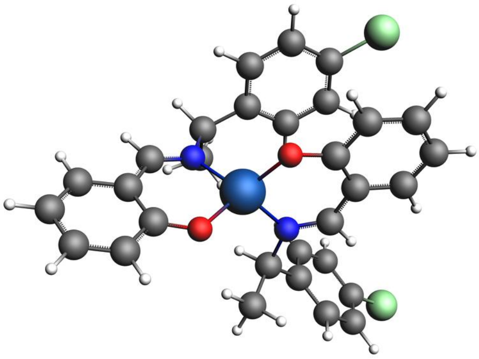

Δ-(R)-conf 36

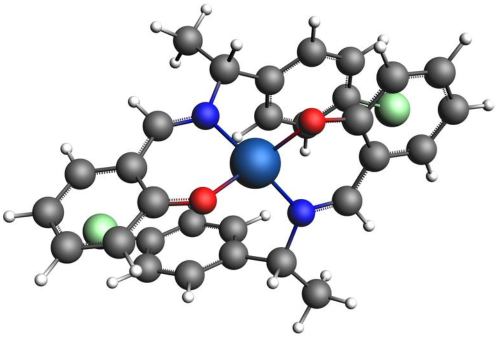

Δ-(R)-conf 38

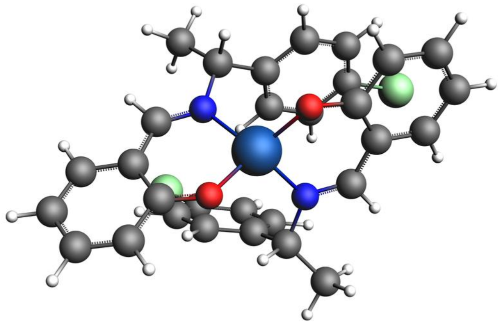

Δ-(R)-conf 44

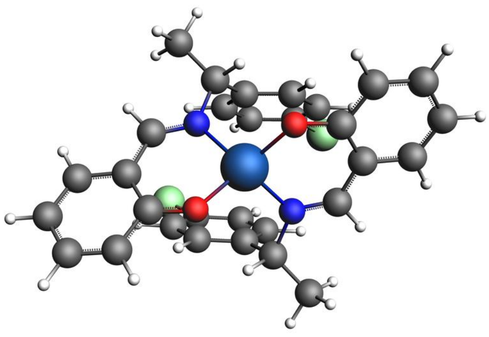

Supplement: Supplementary file 2 [file CP-028-D6CP02625G-s001.zip › SI files/si_plots/delta_R_confs_cropped_grid_same_crop_3col.pdf]
