# Supplementary material for: Vibrational circular dichroism enhancement in conformationally flexible transition metal complexes
Source: Phys Chem Chem Phys. 2026 Sep 16;28(38):23655–63. doi: 10.1039/d6cp02625g (PMC13580204; doi:10.1039/d6cp02625g)
Supplement: Supplementary file 2 [file CP-028-D6CP02625G-s001.zip › SI files/si_plots/Enhanced_l11_d0_E1_oneplot_SI.pdf]

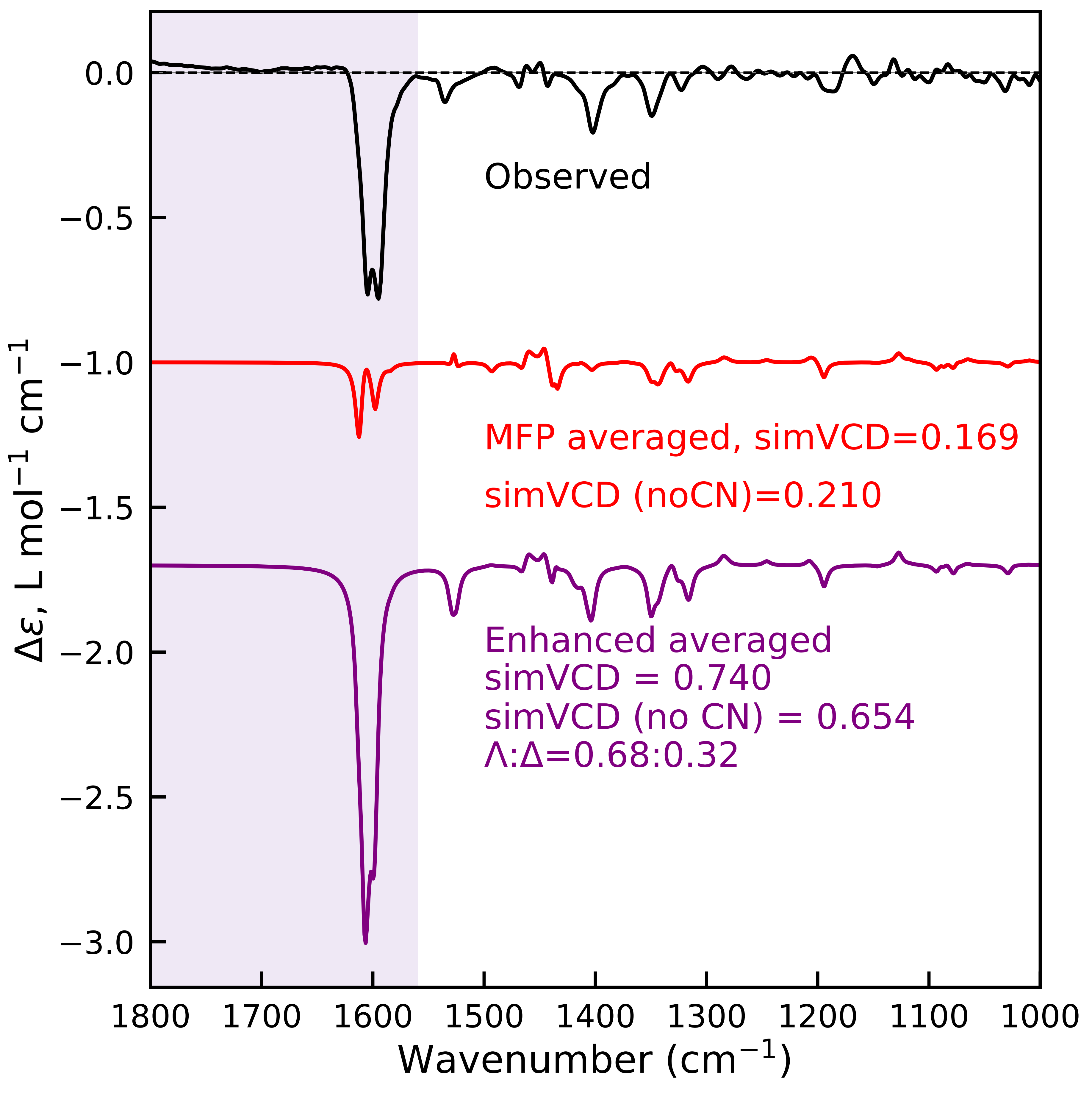

0.0
−0.5
−1.0
−1.5
−2.0
−2.5
−3.0
Δε, L mol⁻¹ cm⁻¹
1800
1700
1600
1500
1400
1300
1200
1100
1000
Wavenumber (cm⁻¹)
Observed
MFP averaged, simVCD=0.169
simVCD (noCN)=0.210
Enhanced averaged
simVCD = 0.740
simVCD (no CN) = 0.654
Λ:Δ=0.68:0.32
