# Supplementary material for: Vibrational circular dichroism enhancement in conformationally flexible transition metal complexes
Source: Phys Chem Chem Phys. 2026 Sep 16;28(38):23655–63. doi: 10.1039/d6cp02625g (PMC13580204; doi:10.1039/d6cp02625g)
Supplement: Supplementary file 2 [file CP-028-D6CP02625G-s001.zip › SI files/si_plots/Enhanced_VCD_nosolv_noCN_E1.pdf]

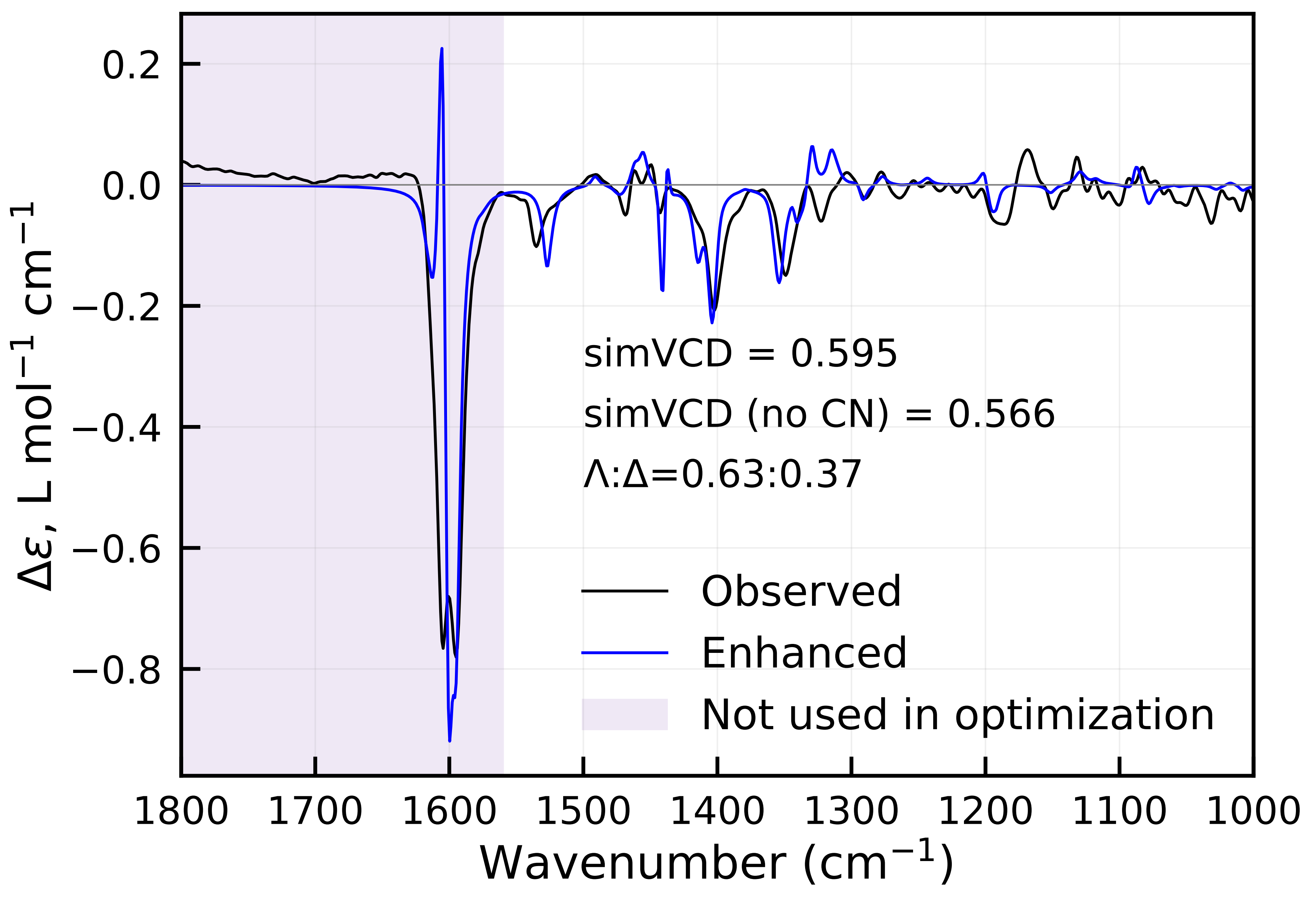

0.2
0.0
−0.2
−0.4
−0.6
−0.8
Δε, L mol⁻¹ cm⁻¹
1800
1700
1600
1500
1400
1300
1200
1100
1000
Wavenumber (cm⁻¹)
simVCD = 0.595
simVCD (no CN) = 0.566
Λ:Δ=0.63:0.37
Observed
Enhanced
Not used in optimization
