# Supplementary material for: Vibrational circular dichroism enhancement in conformationally flexible transition metal complexes
Source: Phys Chem Chem Phys. 2026 Sep 16;28(38):23655–63. doi: 10.1039/d6cp02625g (PMC13580204; doi:10.1039/d6cp02625g)
Supplement: Supplementary file 2 [file CP-028-D6CP02625G-s001.zip › SI files/si_plots/full_enhanced_vcd_full_vs_cluster_reduced_uncertainty_l11freq_E1.pdf]

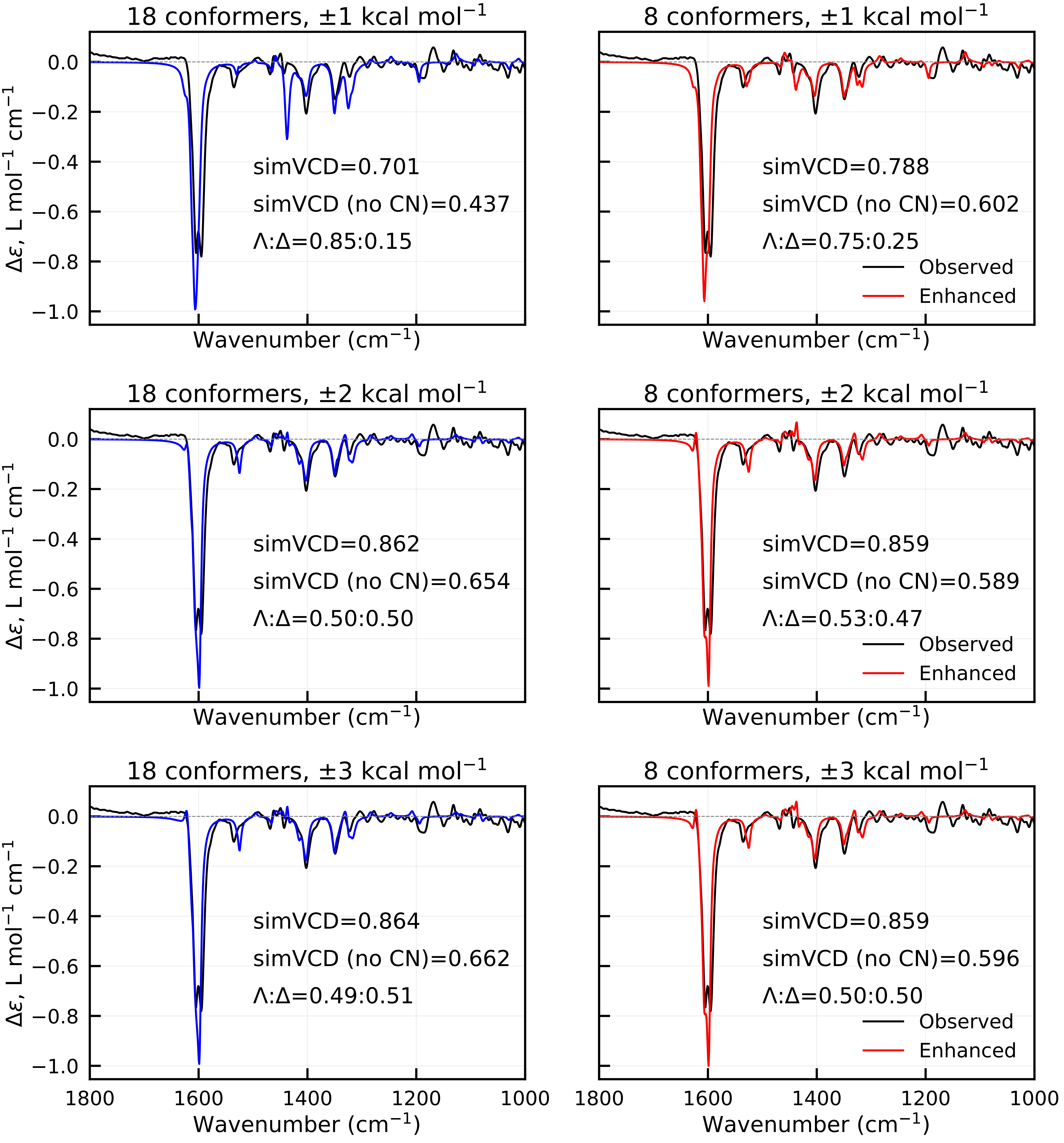

18 conformers, ±1 kcal mol⁻¹
simVCD=0.701
simVCD (no CN)=0.437
Λ:Δ=0.85:0.15
8 conformers, ±1 kcal mol⁻¹
simVCD=0.788
simVCD (no CN)=0.602
Λ:Δ=0.75:0.25
18 conformers, ±2 kcal mol⁻¹
simVCD=0.862
simVCD (no CN)=0.654
Λ:Δ=0.50:0.50
8 conformers, ±2 kcal mol⁻¹
simVCD=0.859
simVCD (no CN)=0.589
Λ:Δ=0.53:0.47
18 conformers, ±3 kcal mol⁻¹
simVCD=0.864
simVCD (no CN)=0.662
Λ:Δ=0.49:0.51
8 conformers, ±3 kcal mol⁻¹
simVCD=0.859
simVCD (no CN)=0.596
Λ:Δ=0.50:0.50
Observed
Enhanced
Δε, L mol⁻¹ cm⁻¹
Wavenumber (cm⁻¹)
