# Supplementary material for: Vibrational circular dichroism enhancement in conformationally flexible transition metal complexes
Source: Phys Chem Chem Phys. 2026 Sep 16;28(38):23655–63. doi: 10.1039/d6cp02625g (PMC13580204; doi:10.1039/d6cp02625g)
Supplement: Supplementary file 2 [file CP-028-D6CP02625G-s001.zip › SI files/si_plots/IR_conformers_6kcal_simir.pdf]

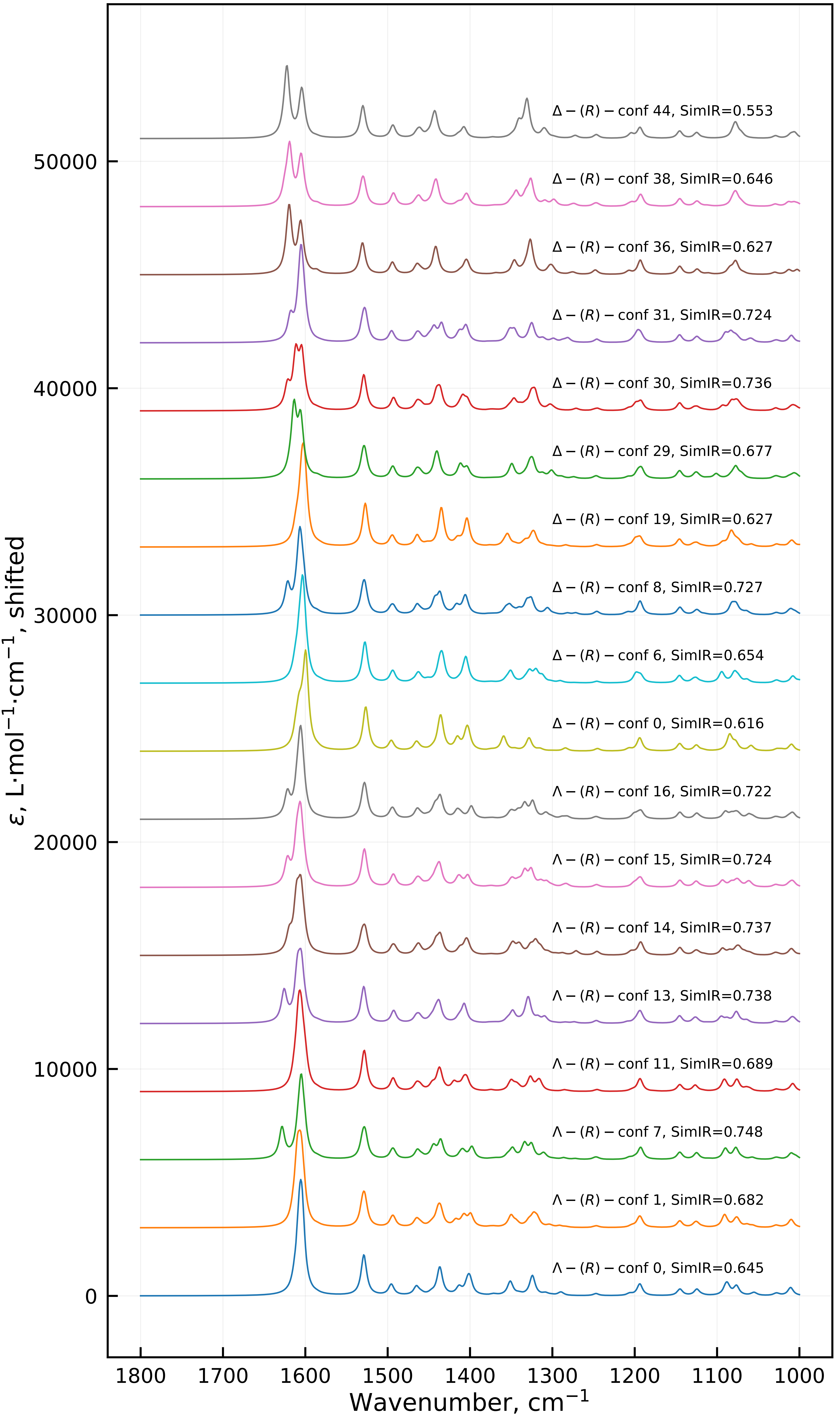
Δ − (R) − conf 44, SimIR=0.553
Δ − (R) − conf 38, SimIR=0.646
Δ − (R) − conf 36, SimIR=0.627
Δ − (R) − conf 31, SimIR=0.724
Δ − (R) − conf 30, SimIR=0.736
Δ − (R) − conf 29, SimIR=0.677
Δ − (R) − conf 19, SimIR=0.627
Δ − (R) − conf 8, SimIR=0.727
Δ − (R) − conf 6, SimIR=0.654
Δ − (R) − conf 0, SimIR=0.616
Λ − (R) − conf 16, SimIR=0.722
Λ − (R) − conf 15, SimIR=0.724
Λ − (R) − conf 14, SimIR=0.737
Λ − (R) − conf 13, SimIR=0.738
Λ − (R) − conf 11, SimIR=0.689
Λ − (R) − conf 7, SimIR=0.748
Λ − (R) − conf 1, SimIR=0.682
Λ − (R) − conf 0, SimIR=0.645
ε, L·mol⁻¹·cm⁻¹, shifted
50000
40000
30000
20000
10000
0
1800
1700
1600
1500
1400
1300
1200
1100
1000
Wavenumber, cm⁻¹
