# Supplementary material for: Vibrational circular dichroism enhancement in conformationally flexible transition metal complexes
Source: Phys Chem Chem Phys. 2026 Sep 16;28(38):23655–63. doi: 10.1039/d6cp02625g (PMC13580204; doi:10.1039/d6cp02625g)

Λ-(R)-conf 0

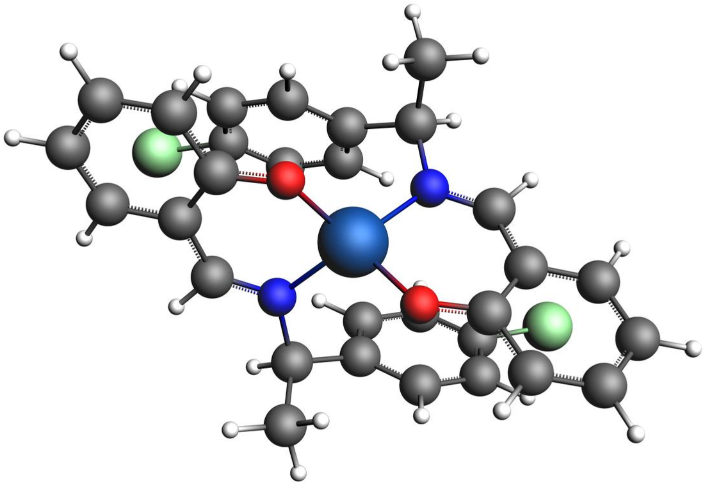

Λ-(R)-conf 1

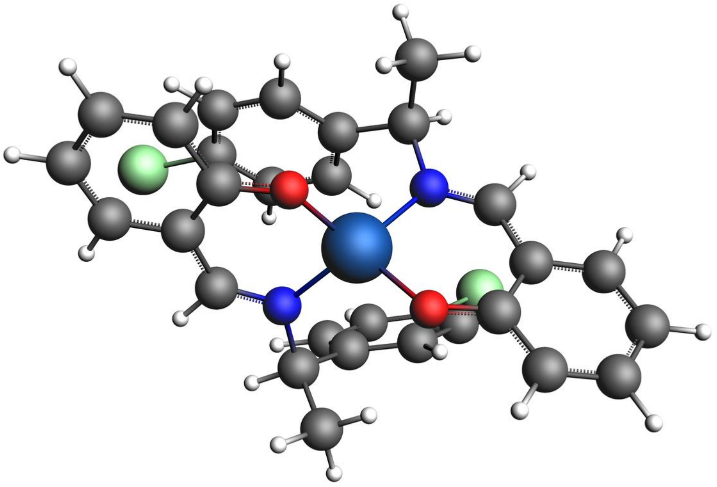

Λ-(R)-conf 7

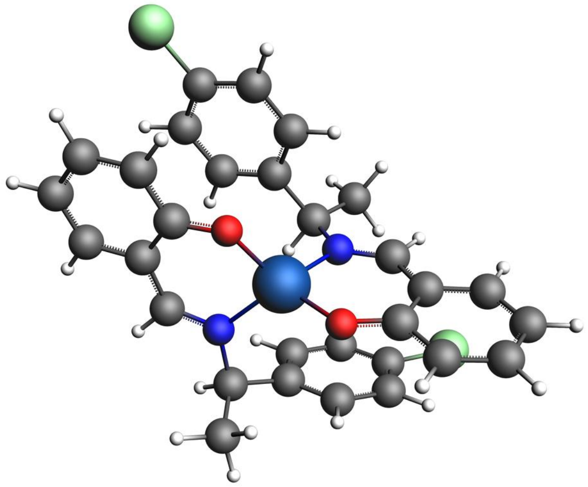

Λ-(R)-conf 11

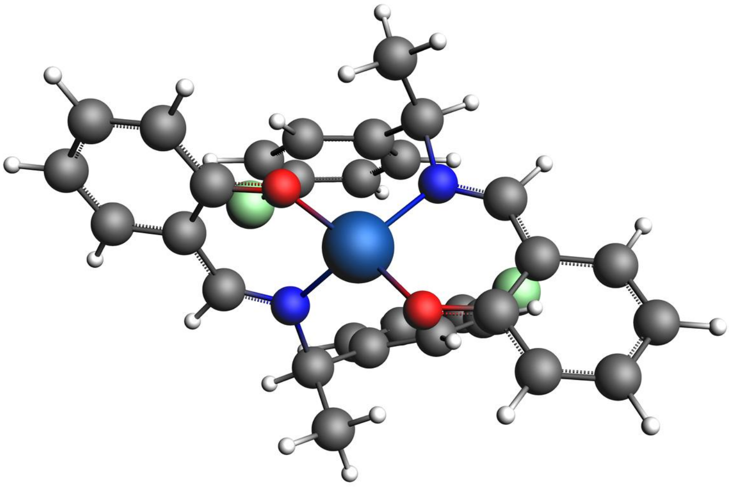

Λ-(R)-conf 13

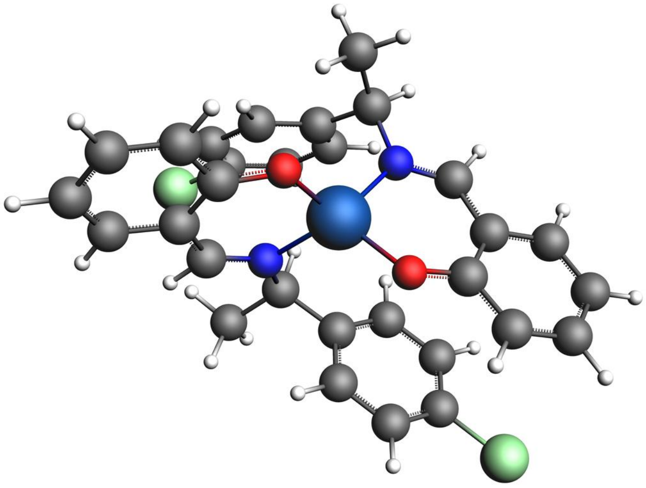

Λ-(R)-conf 14

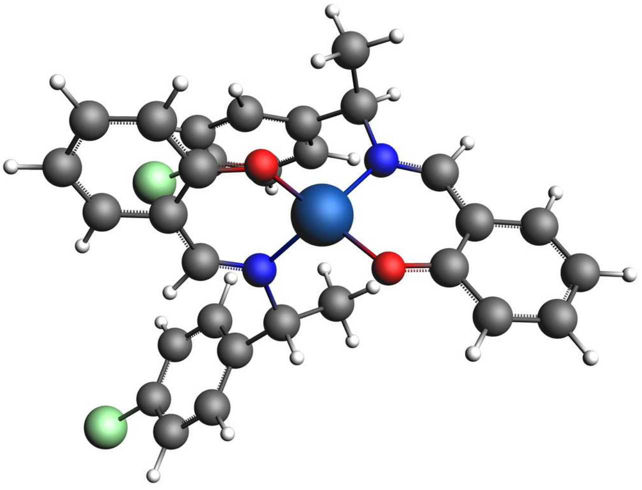

Λ-(R)-conf 15

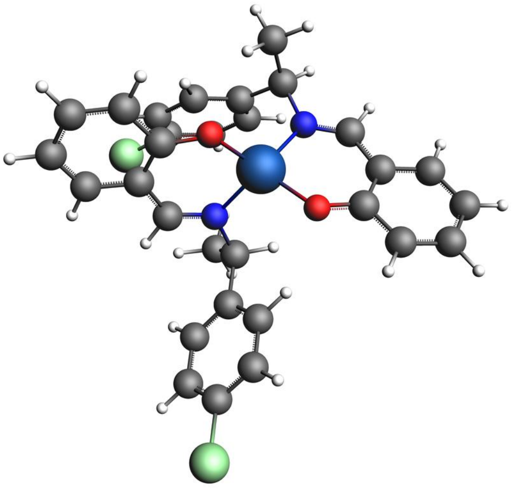

Λ-(R)-conf 16

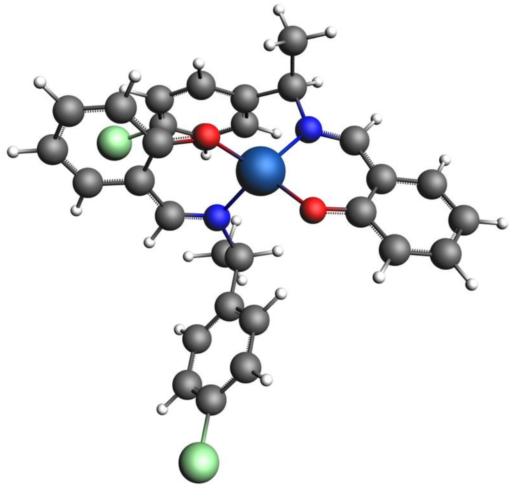

Supplement: Supplementary file 2 [file CP-028-D6CP02625G-s001.zip › SI files/si_plots/lambda_confs.pdf]
