# Supplementary material for: Vibrational circular dichroism enhancement in conformationally flexible transition metal complexes
Source: Phys Chem Chem Phys. 2026 Sep 16;28(38):23655–63. doi: 10.1039/d6cp02625g (PMC13580204; doi:10.1039/d6cp02625g)
Supplement: Supplementary file 2 [file CP-028-D6CP02625G-s001.zip › SI files/si_plots/noCN_enhanced_vcd_full_vs_cluster_reduced_uncertainty_l11freq_E1.pdf]

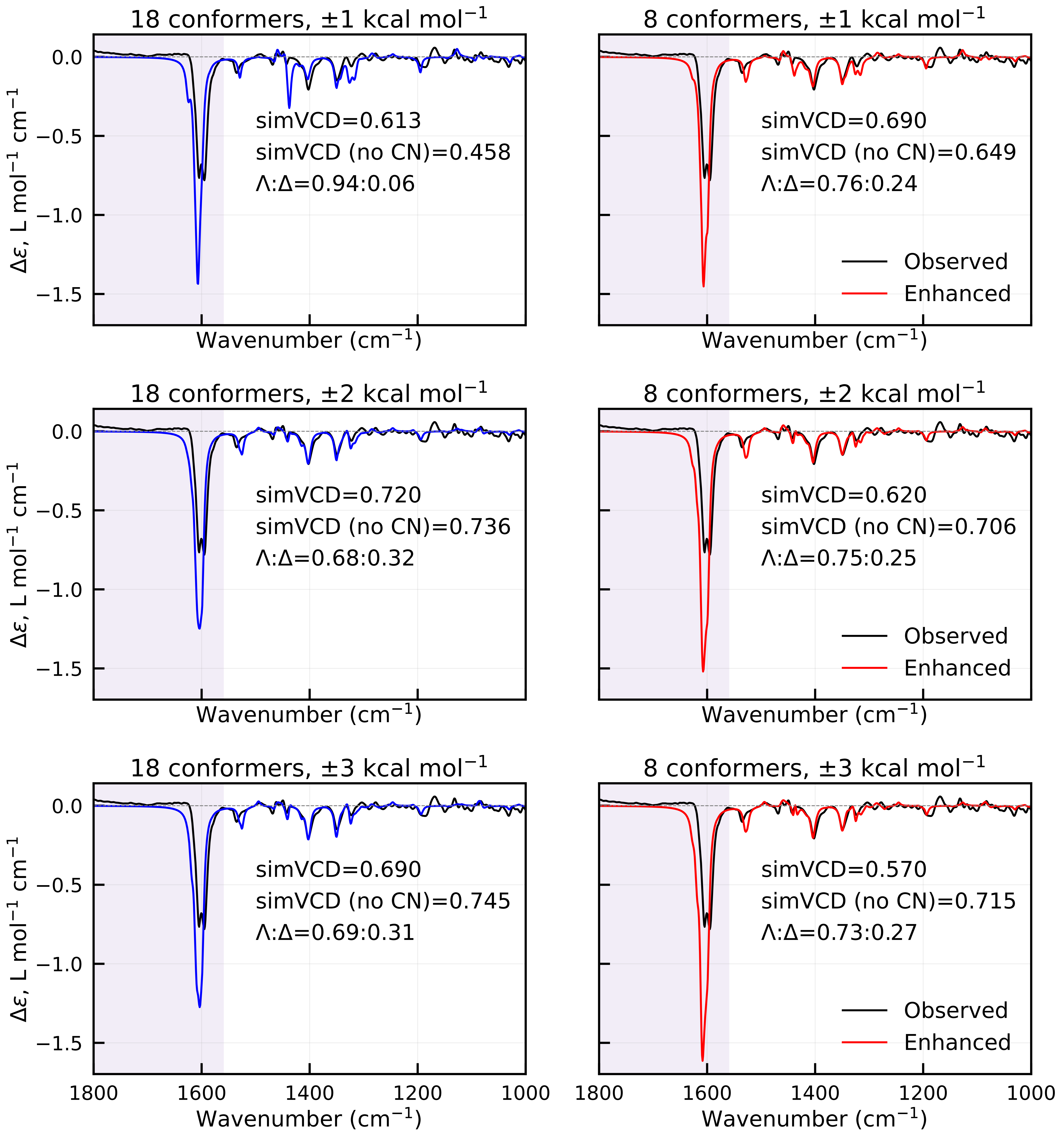
18 conformers, ±1 kcal mol^{-1}
simVCD=0.613
simVCD (no CN)=0.458
Λ:Δ=0.94:0.06
8 conformers, ±1 kcal mol^{-1}
simVCD=0.690
simVCD (no CN)=0.649
Λ:Δ=0.76:0.24
18 conformers, ±2 kcal mol^{-1}
simVCD=0.720
simVCD (no CN)=0.736
Λ:Δ=0.68:0.32
8 conformers, ±2 kcal mol^{-1}
simVCD=0.620
simVCD (no CN)=0.706
Λ:Δ=0.75:0.25
18 conformers, ±3 kcal mol^{-1}
simVCD=0.690
simVCD (no CN)=0.745
Λ:Δ=0.69:0.31
8 conformers, ±3 kcal mol^{-1}
simVCD=0.570
simVCD (no CN)=0.715
Λ:Δ=0.73:0.27
Observed
Enhanced
Δε, L mol^{-1} cm^{-1}
Wavenumber (cm^{-1})
0.0
−0.5
−1.0
−1.5
1800
1600
1400
1200
1000
