# Supplementary material for: Vibrational circular dichroism enhancement in conformationally flexible transition metal complexes
Source: Phys Chem Chem Phys. 2026 Sep 16;28(38):23655–63. doi: 10.1039/d6cp02625g (PMC13580204; doi:10.1039/d6cp02625g)
Supplement: Supplementary file 2 [file CP-028-D6CP02625G-s001.zip › SI files/si_plots/OCH3_subs_enhanced_l11_d0_E1-2.pdf]

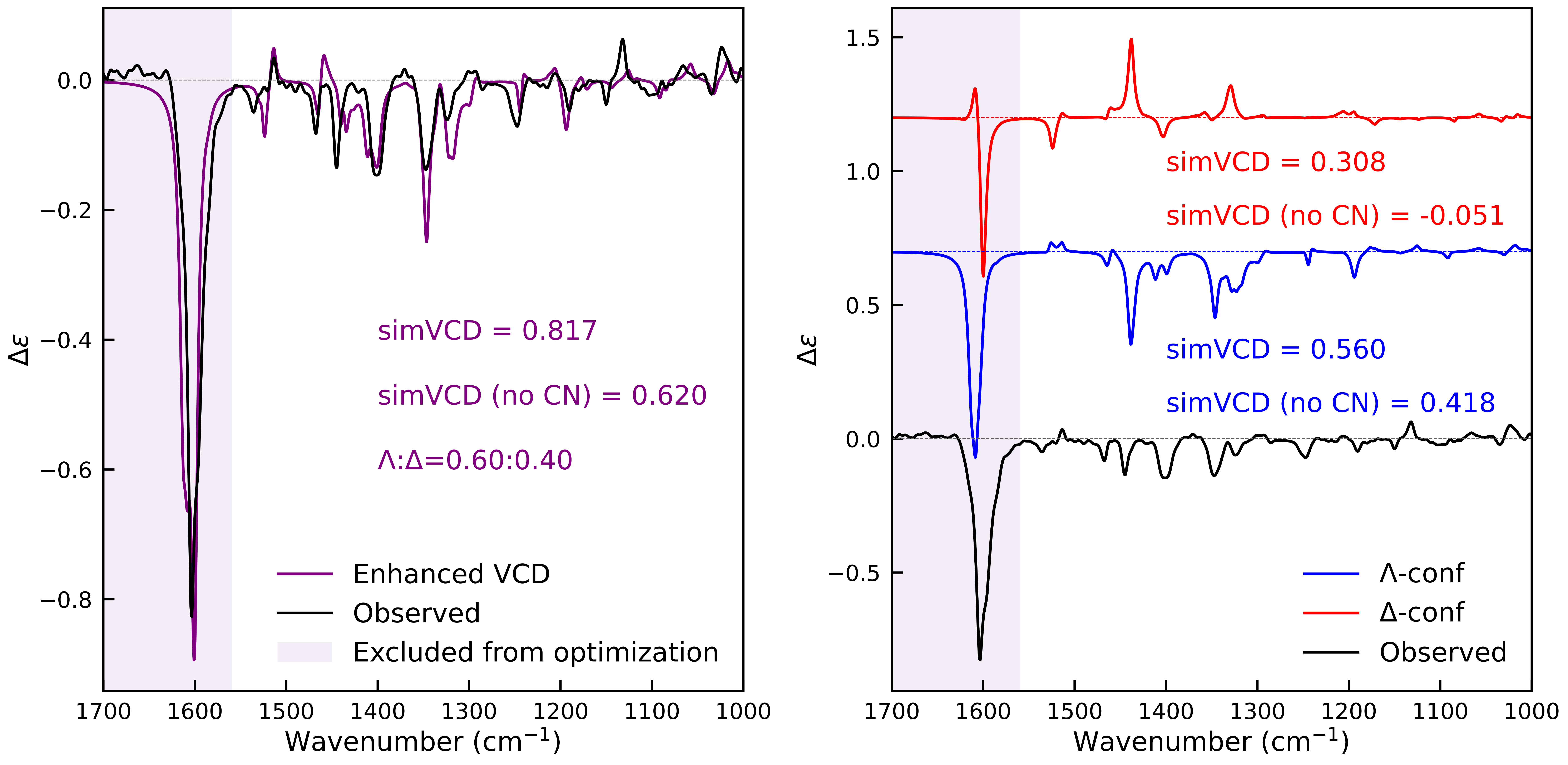

0.0
−0.2
−0.4
−0.6
−0.8
Δε
simVCD = 0.817
simVCD (no CN) = 0.620
Λ:Δ=0.60:0.40
Enhanced VCD
Observed
Excluded from optimization
1700
1600
1500
1400
1300
1200
1100
1000
Wavenumber (cm−1)
1.5
1.0
0.5
0.0
−0.5
Δε
simVCD = 0.308
simVCD (no CN) = -0.051
simVCD = 0.560
simVCD (no CN) = 0.418
Λ-conf
Δ-conf
Observed
1700
1600
1500
1400
1300
1200
1100
1000
Wavenumber (cm−1)
