# Supplementary material for: Vibrational circular dichroism enhancement in conformationally flexible transition metal complexes
Source: Phys Chem Chem Phys. 2026 Sep 16;28(38):23655–63. doi: 10.1039/d6cp02625g (PMC13580204; doi:10.1039/d6cp02625g)
Supplement: Supplementary file 2 [file CP-028-D6CP02625G-s001.zip › SI files/si_plots/VCD_MFP_conformers_6kcal_simvcd.pdf]

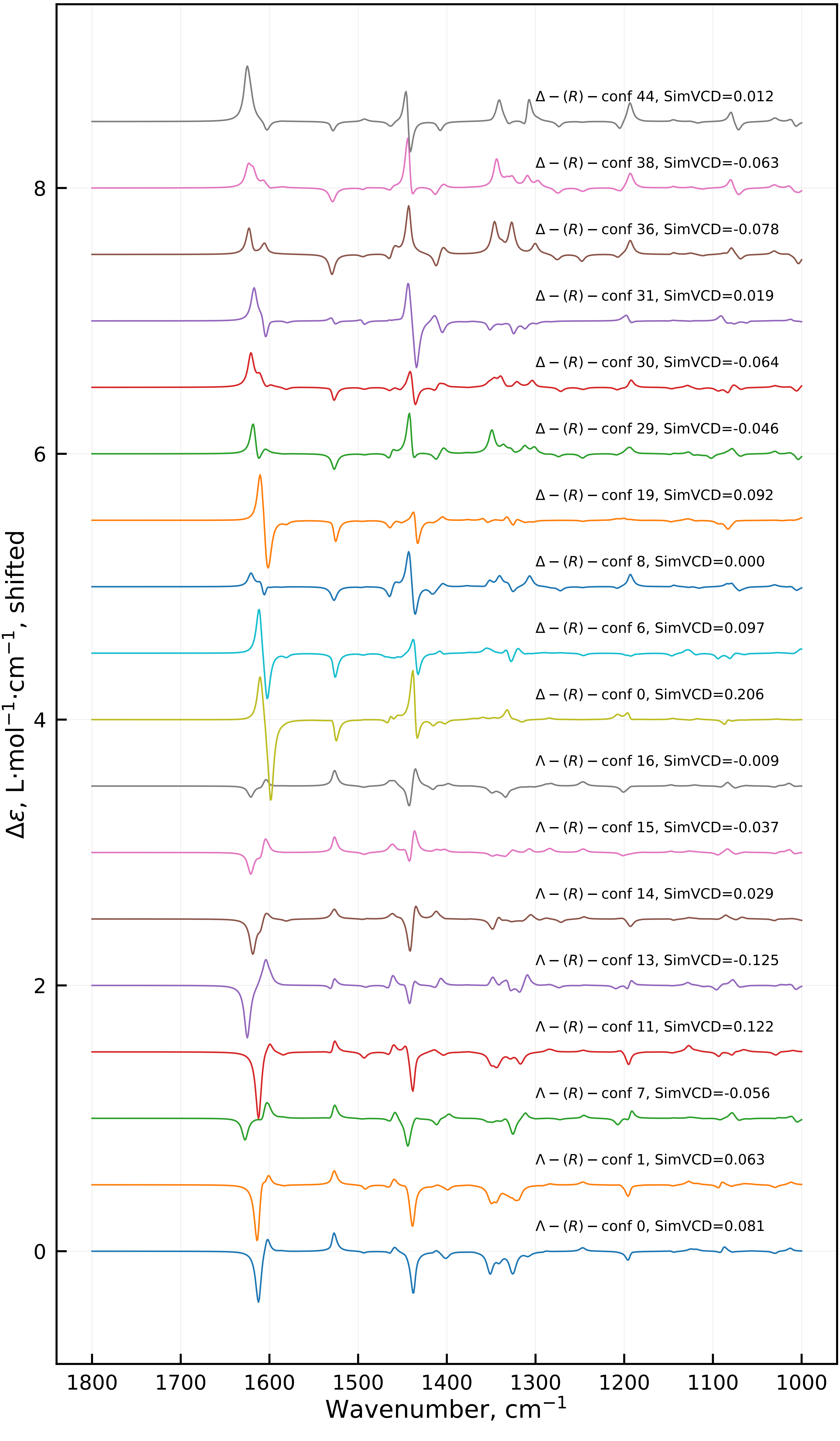

Δ − (R) − conf 44, SimVCD=0.012
Δ − (R) − conf 38, SimVCD=-0.063
Δ − (R) − conf 36, SimVCD=-0.078
Δ − (R) − conf 31, SimVCD=0.019
Δ − (R) − conf 30, SimVCD=-0.064
Δ − (R) − conf 29, SimVCD=-0.046
Δ − (R) − conf 19, SimVCD=0.092
Δ − (R) − conf 8, SimVCD=0.000
Δ − (R) − conf 6, SimVCD=0.097
Δ − (R) − conf 0, SimVCD=0.206
Λ − (R) − conf 16, SimVCD=-0.009
Λ − (R) − conf 15, SimVCD=-0.037
Λ − (R) − conf 14, SimVCD=0.029
Λ − (R) − conf 13, SimVCD=-0.125
Λ − (R) − conf 11, SimVCD=0.122
Λ − (R) − conf 7, SimVCD=-0.056
Λ − (R) − conf 1, SimVCD=0.063
Λ − (R) − conf 0, SimVCD=0.081
8
6
4
2
0
Δε, L·mol⁻¹·cm⁻¹, shifted
1800
1700
1600
1500
1400
1300
1200
1100
1000
Wavenumber, cm⁻¹
